# Antifungal Potential of *Piper*-Derived Essential Oils and Key Constituents on *Moniliophthora roreri*, the Causal Agent of Moniliasis in Cacao (*Theobroma cacao* L.)

**DOI:** 10.3390/plants14162514

**Published:** 2025-08-13

**Authors:** Natalia V. Delgado-Bogotá, Oscar J. Patiño-Ladino, Juliet A. Prieto-Rodríguez

**Affiliations:** 1 Departamento de Química, Facultad de Ciencias, Universidad Nacional de Colombia, Sede Bogotá, Bogotá 111321, Colombia; ndelgadob@unal.edu.co; 2 Departamento de Química, Facultad de Ciencias, Pontificia Universidad Javeriana, Bogotá 110231, Colombia; juliet.prieto@javeriana.edu.co

**Keywords:** *Piper*, essential oils, *Moniliophthora roreri*, moniliasis, vapor-phase diffusion antifungal activity, phenylpropanoids

## Abstract

*Moniliophthora roreri*, the causal agent of moniliasis, severely affects cacao production in Latin America, and sustainable control alternatives remain limited. This study aimed to evaluate the antifungal potential of essential oils (EOs) from *Piper* species and selected volatile compounds against *M. roreri*. A total of 34 EOs obtained by steam distillation were assessed for mycelial growth inhibition under fumigation conditions. The most active EOs (≥60% inhibition) were chemically characterized by GC-MS, and their median inhibitory concentrations (IC_50_) were determined. Additionally, 40 structurally diverse volatile compounds were selected and evaluated for their antifungal activity to identify the main contributors and explore structure–activity relationships. Most of the active EOs exhibited a high proportion of phenylpropanoids and oxygenated monoterpenoids, with IC_50_ values ranging from 0.58 to 184.27 µL·L^−1^; the most active were those from *P. holtonii* and *P. aduncum*. Among the 28 most active compounds, IC_50_ values ranged from 0.48 to 109.81 µL·L^−1^; the most potent were myristicin and dillapiole. The most potent molecules were phenylpropanoids bearing methoxy and methylenedioxy groups, followed by oxygenated monoterpenoids and long-chain ketones. This is the first report of antifungal activity against *M. roreri* for most of the evaluated EOs and all tested compounds, highlighting the potential of the *Piper* genus as a source of natural alternatives for sustainable disease management in cacao cultivation.

## 1. Introduction

*Moniliophthora roreri* (Cif.) H.C. Evans (Marasmiaceae) is a highly aggressive Basidiomycete fungus and the causal agent of frosty pod rot (FPR), one of the most devastating diseases affecting cacao (*Theobroma cacao* L.) in Latin America [1,2,3]. This pathogen primarily infects the fruit, leading to total or partial pod rot, with symptoms including premature ripening, hypertrophy, deformities, subepidermal oily spots, brown lesions covered by a cream-colored mycelial mat, wilting, and fruit necrosis, depending on developmental stage and environmental conditions. [1,4]. Native to Colombia, *M. roreri* has spread to major cacao-producing regions across Central and South America [3,5,6]. Yield losses caused by this pathogen can reach up to 90% under warm, humid climates with annual rainfall between 1200 and 4000 mm, making it one of the main drivers of crop abandonment among smallholder farmers [1,4,7].

Control strategies have focused on quarantine measures and cultural practices, often supplemented by the application of synthetic fungicides [4,8,9]. However, the limited efficacy of some commercial products, combined with environmental and health concerns and the emergence of resistant fungal strains, underscores the need for sustainable and safe alternatives [10,11,12]. Given this scenario, it becomes imperative to explore sustainable and safe alternatives for the control of *M. roreri* that can reduce dependence on synthetic products and mitigate their associated adverse effects. In this context, plants represent a promising source of bioactive compounds for crop protection [13,14,15].

Essential oils (EOs) are complex mixtures of volatile secondary metabolites derived from plants, which have shown promising activity against agricultural pests and pathogens [6,16,17,18,19,20]. In general, essential oils have been reported to exert multiple antifungal effects, including (1) disruption of membrane function and structure through the denaturation of membrane proteins; (2) inhibition of cellular respiration and alteration in membrane permeability at low concentrations; (3) severe membrane damage, homeostatic imbalance, and cell death at higher concentrations; (4) precipitation of cellular proteins and inhibition of key enzymes involved in energy production and biosynthesis of structural components; (5) increased membrane permeability leading to leakage of vital intracellular constituents; and (6) inhibition of mycotoxin production [21,22,23,24,25]. Despite this potential, research on the use of EOs against *M. roreri* remains limited, often restricted to preliminary screenings, with little exploration of their major constituents or mechanisms of action [26,27,28,29,30].

Among plant genera of interest, *Piper* (Piperaceae) has received growing attention due to its chemical diversity and the antifungal properties of many of its species [2,10,31,32,33]. Prior studies have reported antifungal activity of *P. aduncum* and *P. auritum* EOs against *M. roreri* [26,34,35]. Additionally, ethanolic extracts of *P. peltatum* have demonstrated significant antifungal effects, and a broader screening of ethanolic extracts from eleven *Piper* species identified *P. asperiusculum*, *P. grande*, *P. statarium*, *P. artanthe*, and *P. nigrum* as particularly effective, with IC_50_ values for mycelial growth inhibition ranging from 692 ppm to below 125 ppm [36,37]. Studies on *P. pesaresanum* and *P. ceanothifolium* revealed the antifungal properties of their extracts and some chemical constituents against this phytopathogen and allowed for the establishment of preliminary structure–activity relationships. These investigations highlighted the inhibitory activity of alkylphenols, chalcones, and prenylated derivatives of benzoic acid [2,10]. This study evaluates the antifungal potential of 34 *Piper* essential oils and 40 structurally diverse volatile compounds against *M. roreri*, characterizes their chemical profiles, and explores structure–activity relationships to support the development of natural vapor-phase control strategies.

## 2. Results and Discussion

### 2.1. Screening of Antifungal Activity of Piper EOs Against M. roreri

**Figure 1:**
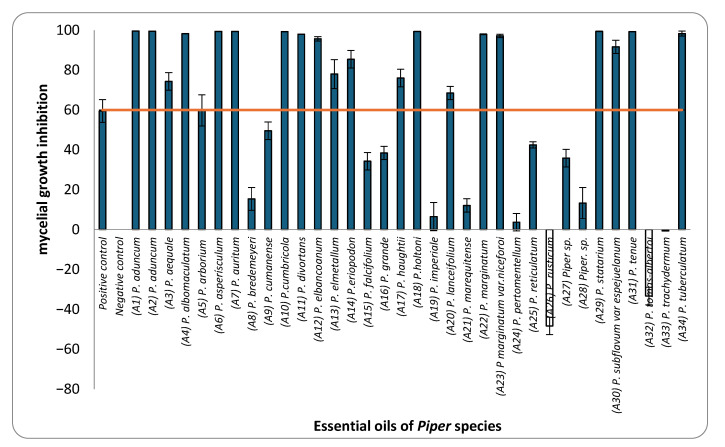
Mycelial growth inhibition (MGI) of *M. roreri* by essential oils from *Piper* species at a concentration of 183 µL/L of air.

This study assessed the mycelial growth inhibition (MGI) of *M. roreri* by 34 EOs obtained from *Piper* species, using a concentration of 183 µL/L of air, evaluated through a vapor-phase diffusion assay specifically chosen due to the volatile and hydrophobic nature of essential oil and their constituents, which limits their effectiveness in conventional agar-based methods such as dilution or disc diffusion. These traditional techniques rely on diffusion through aqueous media, where volatile substances may not dissolve efficiently and tend to evaporate during incubation, reducing their contact with the fungal target. In contrast, the vapor-phase diffusion assay enables a controlled vapor-phase exposure, allowing for accurate assessment of antifungal activity by calculating the concentration of EO in the headspace (µL/L of air), which more closely reflects real conditions of application for volatile agents. The results revealed that 20 of these EOs inhibited more than 60% of the radial fungal growth (Figure 1). Among these, the EOs from *P. holtoni*, *P. aduncum*, *P. asperiusculum*, *P. tenue*, *P. auritum*, and *P. statarium* stood out for their strong antifungal activity, each exhibiting inhibition rates above 99%. Additionally, EOs from *P. tuberculatum*, *P. divortans*, *P. marginatum*, and *P. albomaculatum* showed inhibition rates exceeding 90%. In contrast, moderate antifungal activity (60–89%) was observed for EOs from *P. elmetanum*, *P. lanceifolium*, *P. eriopodon*, *P. aequale*, and *P. haugtii*, while others, such as those from *P. imperiale*, *P. pertomentellum*, *P. grande*, *P. arborium*, *Piper* sp., and *P. marequitense*, exhibited considerably lower efficacy. Interestingly, essential oils from *P. rusticum* and *P. tomas-albertoi* promoted mycelial growth instead of inhibiting it. Similar stimulatory effects have been reported in the literature, where certain essential oils or their volatile constituents enhance sporulation or growth in fungi under sublethal chemical stress. For example, Hountondji, 2006 [38], showed that citrus essential oils increased sporulation in *Phaeoramularia angolensis*, while other studies suggest that some volatiles may interfere with fungal regulatory pathways, triggering adaptive or compensatory responses [39,40,41].

Among the 34 EOs tested, only those from *P. aduncum*, *P. cumanense*, and *P. auritum* had been previously reported to inhibit *M. roreri*. In earlier studies, EO from *P. aduncum*, extracted from shoots, leaves, and inflorescences, significantly inhibited fungal growth through both contact and fumigation, particularly at volumes of 15 and 30 µL [34]. Similarly, *P. cumanense* demonstrated complete inhibition in contact assays at concentrations near 52 mg/mL [35]. For *P. auritum*, a partial reduction in fungal growth was reported, although the specific concentrations used were not provided [26]. Although research on the antifungal activity of *Piper* EOs against *M. roreri* remains limited, prior studies have documented their efficacy against other cacao-associated phytopathogens. For instance, EOs from *P. chaba*, *P. callosum*, *P. enckea*, *P. divaricatum, P. marginatum*, *P. marginatum var. anisatum*, and *P. dilatatum* have shown activity against *Fusarium solani*, *Phytophthora palmivora* and *P. capsici*, pathogens that share ecological niches with *M. roreri* [42,43,44]. Within this context, the present findings significantly expand the existing knowledge base by providing the first report of vapor-phase diffusion activity against *M. roreri* for 31 of the 34 EOs evaluated.

Based on these results, EOs exhibiting ≥ 60% inhibition were selected for dose–response assays to determine their IC_50_ values. This quantitative approach enabled a comparative ranking of the fungicidal potency of the selected EOs and contributed to a more comprehensive understanding of their activity against the pathogen. Table 1 presents the IC_50_ values in ascending order, from the most to the least active EOs. The IC_50_ values ranged from 0.58 to 184.27 µL L^−1^ of air. The one-way analysis of variance (ANOVA) performed on the IC_50_ values of the EOs revealed significant differences (F = 3844.77; *p* < 0.001). Tukey’s post hoc multiple comparison test allowed for the grouping of the EOs into 13 homogeneous subsets, designated with the letters A to M in the corresponding table. Group A included the most active EOs, with IC_50_ values ranging from 0.58 to 2.05 µL L^−1^, while group M comprised the least active EO, with an IC_50_ of 184.27 µL L^−1^. Notably, the EOs from *P. holtoni* (A18), *P. aduncum* (A1 and A2), and *P. statarium* (A29) exhibited IC_50_ values below 3 µL L^−1^, classifying them as highly active. In contrast, the EOs from *P. albomaculatum*, *P. elmetanum*, and *P. lanceifolium* had IC_50_ values above 100 µL L^−1^, indicating limited efficacy.

### 2.2. Chemical Characterization of EOs with Potential Activity Against M. roreri

The chemical composition of the 20 bioactive EOs was analyzed using gas chromatography–mass spectrometry (GC-MS) with orthogonal polarity columns. This approach allowed for the identification of 122 compounds, which together accounted for more than 90% of the total composition of the analyzed oils. Table 2 summarizes the major constituents of each EO (defined as those exceeding 3% relative area). Detailed compositions obtained with both DB-5MS and HP-INNOWax columns are provided in Table S3.

**Figure 2:**
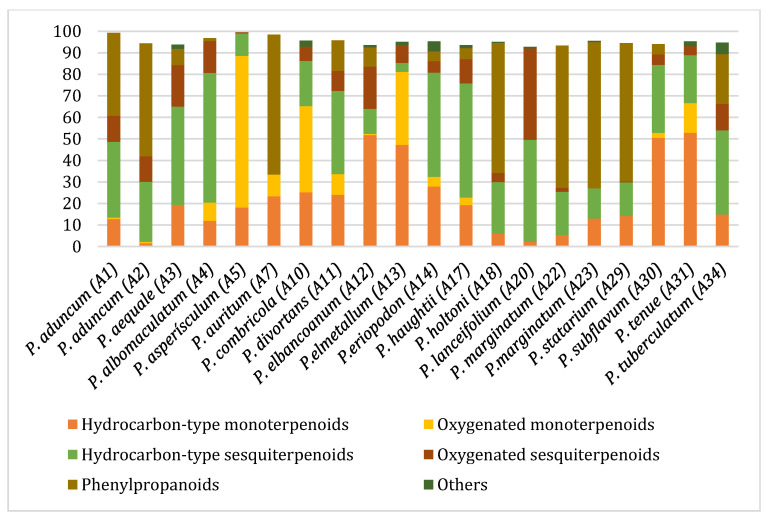
Distribution of major chemical classes in EOs from *Piper* species with antifungal activity against *M. roreri*.

Figure 2 illustrates the relative distribution of the main chemical classes identified in the EOs, including hydrocarbon and oxygenated sesquiterpenoids, hydrocarbon and oxygenated monoterpenoids, and, to a lesser extent, phenylpropanoids. The abundance of these chemical groups varied considerably among the different *Piper* species. EOs from *P. subflavum*, *P. asperiusculum*, and *P. aequale* were characterized by a predominance of monoterpenes such as α- and β-pinene, limonene, and piperitone. In contrast, *P. huantlii*, *P. eriopodon*, *P. lanceolifolium*, and *P. tuberculatum* exhibited higher levels of sesquiterpenes, with β-caryophyllene, germacrene D, and α-copaene among the most abundant. Phenylpropanoids were the dominant class in the EOs of *P. aduncum*, *P. marginatum*, *P. holtonii*, and *P. auritum*, where major constituents included estragole, dillapiole, apiole, and safrole. Although no definitive trend was established linking a specific chemical class to stronger antifungal effects, EOs with high phenylpropanoid content tended to show enhanced activity against *M. roreri*. These oils also demonstrated substantial structural diversity, with major components such as dillapiole, myristicin, elemicin, apiole, and safrole, which have been previously associated with antifungal activity and are recognized as typical metabolites in various *Piperaceae* species [26,45,46,47,48].

From a chemical perspective, previous studies on *Piper* species have demonstrated that the volatile fractions from aerial parts are predominantly composed of monoterpenoids and sesquiterpenoids, with occasional reports of diterpenes and phenylpropanoids [49]. The chemical profiles observed in this study are largely consistent with the existing literature, though notable interspecific differences were detected. These differences may result from environmental and geographic factors, as well as variations in extraction techniques and analytical methodologies [49,50,51].

For instance, the EO from *Piper aduncum* analyzed in this study exhibited high levels of myristicin and dillapiole, followed by germacrene D and caryophyllene. These results contrast significantly with previously reported profiles, which describe a marked presence of caryophyllene oxide (37.0%), piperitone (23.7%), and viridiflorol (14.5%), as dominant components, in addition to a diverse mixture of monoterpenes and sesquiterpenes such as linalool, camphene, and germacrene D [52]. In another reported chemotype of *P. aduncum*, dillapiole (23.3%) was again predominant, followed by apiole (7.3%), myristicin (5.6%), and viridiflorol (4.1%), supporting the dominance of phenylpropanoids in this species. Nevertheless, other studies have reported substantially higher dillapiole content (73.7%) and only trace levels of the remaining compounds, suggesting that the observed variation in phenolic composition may be attributed to factors such as phenological stage, genetic divergence among populations, or differences in extraction methodologies [49,53,54,55].

The EO of *P. aequale* was primarily composed of E-nerolidol, germacrene B, β-caryophyllene and safrole, reflecting a combination of oxygenated sesquiterpenoids and phenylpropanoids. This composition diverges from previous reports, which highlight α-pinene (12.6%), δ-elemene (19.0%), and cubebol (7.2%) as dominant components, along with limonene (7.9%) and β-pinene (3.3%) as non-major constituents [56]. The EO of *P. auritum* was rich in safrole and camphor, partially consistent with the literature that reports even higher safrole content (93.2%) and detectable myristicin (4.3%) [57]. In *P. asperiusculum*, piperitone was the major constituent, contrasting with prior studies of fresh leaves in which dillapiole (48.5%) and myristicin (11.5%) predominated [58], suggesting that sample condition and plant origin may influence chemical profiles.

In *P. eriopodon*, the EO featured a balanced mixture of β-caryophyllene, α-pinene, β-pinene, α-copaene, germacrene B and apiole, while the presence of α-copaene and β-caryophyllene was consistent with research data [59]. The EO from *P. huaghtii* was characterized by germacrene D, β-caryophyllene and α-copaene, together with cadina-1,4-diene and apiole, in contrast to prior reports where dillapiole (48.2%) was dominant, and β-caryophyllene and piperitone were present at lower levels [60]. In *P. holtonii* EO, the main compounds were apiole, dillapiole and germacrene D; although the dillapiole content was lower than the 64.4% previously described, the consistent presence of germacrene D supports its chemotaxonomic relevance [60].

The EO of *P. lanceolifolium* was dominated by β-caryophyllene, farnesol and eudesmol. In contrast, earlier studies reported safrole (48.3%) as the primary compound, which was not detected in our samples [61]. *P. marginatum* exhibited a profile dominated by estragole and E-anethole, whereas previous research identified γ-asarone (64.5%) as the major metabolite [51]. In *P. subflavum*, high levels of α-pinene and β-pinene were found, accompanied by germacrene D and β-caryophyllene. This monoterpene-rich profile was largely consistent with previous reports, with minor differences such as the presence of apiole (3.4%) [59]. The EO of *P. tuberculatum* was characterized by β-caryophyllene, germacrene D and E-nerolidol. Although β-caryophyllene was also dominant in earlier studies (40.2%), such discrepancies may be attributed to differences in the plant organ or environmental conditions [49].

Several *Piper* EOs have demonstrated significant antifungal activity against phytopathogenic fungi of agricultural importance. For instance, *P. auritum* has shown strong inhibition against *Fusarium* species, with inhibition rates of 75.32% against *F. oxysporum* f. sp. *comiteca*, 86.57% against *F. oxysporum* f. sp. *tequilana*, and 63.36% against *F. solani* f. sp. *comiteca* [62,63]. Similarly, *P. holtonii* EO has been shown to inhibit the mycelial growth of *Colletotrichum acutatum*, *C. gloeosporioides*, and *Botryodiplodia theobromae* at concentrations of 400 µg/mL [64]. In vitro studies on *P. aduncum* reported inhibition rates of up to 94% against *F. solani* and 91% against *Phytophthora* sp. [26]. To the best of our knowledge, this work provides the first chemical characterization of the EOs from eight species: *P. albomaculatum*, *P. cumbricola*, *P. divortans*, *P. elbanoanum*, *P. elmetanum*, *P. marginatum* var. *niceforoi*, *P. statarium*, and *P. tenue*. These new profiles expand the phytochemical knowledge of the genus *Piper* and support its potential in the development of botanical fungicides.

### 2.3. Antifungal Potential of Selected Chemical Constituents Against M. roreri

**Figure 3:**
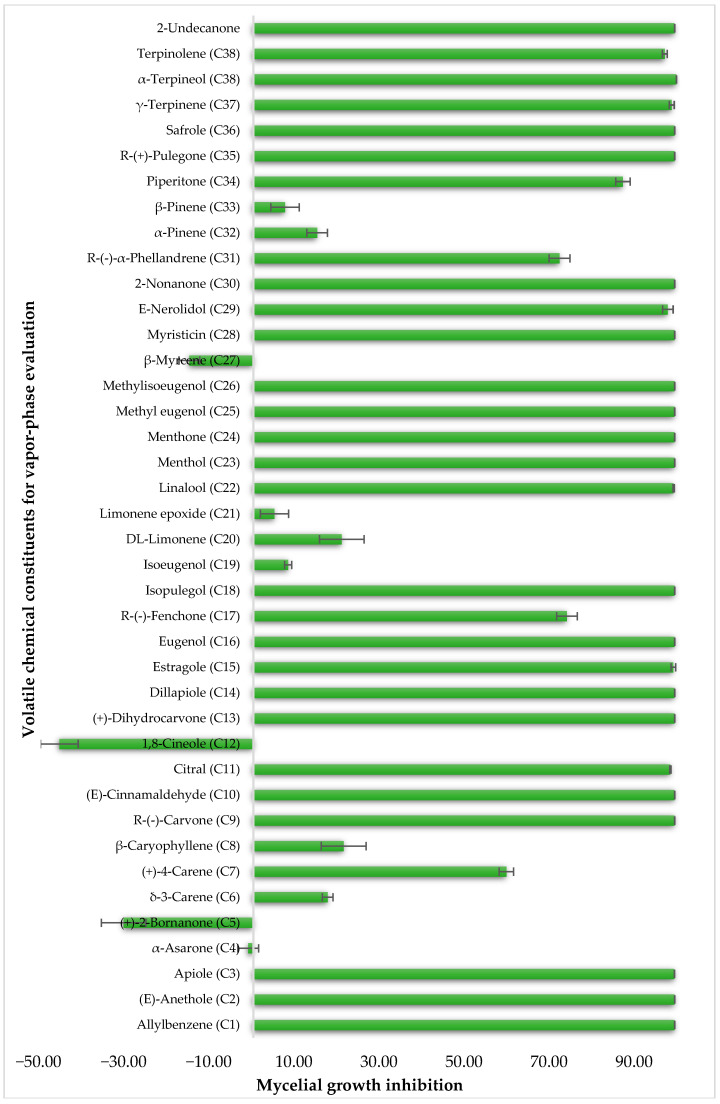
Growth inhibition of *M. roreri* by selected volatile compounds in vapor exposure assays.

To identify individual compounds potentially responsible for the vapor-phase activity observed in the most effective essential oils (EOs), a set of 40 representative substances was selected. This set included 25 compounds detected in the analyzed EOs and 15 additional molecules with relevant structural features for exploring structure–activity relationships. Among the selected compounds were 23 monoterpenoids, 13 phenylpropanoids, 2 sesquiterpenoids, and 2 aliphatic ketones, labeled from C1 to C40 (Table S2). Fumigation-based mycelial growth inhibition (MGI) assays were conducted at a maximum concentration of 122 µL·L^−1^ of air, revealing substantial variability in antifungal responses, with inhibition values ranging from −46.65% to 100%. A total of 28 compounds inhibited more than 60% of *M. roreri* growth (Figure 3). The most active compounds belonged primarily to the classes of phenylpropanoids, oxygenated monoterpenoids, and aliphatic ketones. Among the compounds that achieved over 90% inhibition, all shared the presence of oxygenated groups such as alcohols, ketones, methylenedioxy moieties, or conjugated double bonds inside chains. This study represents the first report of vapor-phase diffusion antifungal activity against *M. roreri* for all tested compounds.

For the 28 promising compounds, median inhibitory concentration (IC_50_) values were determined, and the results are presented in Table 3. A broad range of antifungal potency was observed, with IC_50_ values ranging from 0.48 to 109.8 µL·L^−1^ of air. The one-way analysis of variance (ANOVA) performed on the IC_50_ values of the bioactive compounds revealed significant differences among the evaluated compounds (F = 3844.77; *p* < 0.001). Tukey’s post hoc multiple comparison test allowed for the grouping of the compounds into 13 homogeneous subsets, designated with the letters A to K in the corresponding table. Group A included the most active compounds, with IC_50_ values of less than 1 µL L^−1^, while group K comprised the least active compounds, with an IC_50_ greater than 100 µL L^−1^. The most active compounds were predominantly phenylpropanoids and oxygenated monoterpenoids, characterized by functional groups such as methylenedioxy-substituted aromatic rings, methoxy groups, α,β-unsaturated aldehydes, or tertiary alcohols. Myristicin and dillapiole were particularly notable for their high efficacy, with IC_50_ values below 1 µL·L^−1^, suggesting a key role for these structural motifs in inhibiting mycelial growth. In contrast, the compounds with the lowest activity were mainly hydrocarbon monoterpenes such as γ-terpinene and α-phellandrene.

Although none of the evaluated compounds had been previously reported as active against *M. roreri*, some have been studied in other fungal models. Myristicin, for example, has demonstrated potent antifungal activity in nutmeg essential oil, with inhibition rates exceeding 80% against *Fusarium oxysporum*, *Aspergillus flavus*, and *A. ochraceus* at concentrations between 0.1% and 0.3% [65,66]. It has also been shown to enhance the toxicity of other compounds, such as xanthotoxin, through inhibition of fungal microsomal enzymes [67]. Dillapiole has likewise shown strong antifungal effects, including inhibition of basidiospore germination in *Crinipellis perniciosa* at concentrations as low as 0.6–1.0 ppm [45], and selective inhibition of aflatoxin G1 production in *Aspergillus parasiticus* (IC_50_ = 0.15 μM) [68]. Structurally related compounds such as apiole and myristicin have also shown antifungal activity, with reported IC_50_ values of 0.24 and 3.5 μM, respectively.

With respect to oxygenated monoterpenoids, previous studies have documented antifungal activity for compounds such as citral, linalool, eugenol, methyl eugenol, 1,8-cineole, and α-phellandrene, although these have typically been tested at higher concentrations than those used in the present study [69]. Most *Piper* species are known to produce essential oils with a high proportion of phenylpropanoids. In this context, the results reported here support the hypothesis that these compounds contribute substantially to the antifungal activity observed in the vapor phase against *M. roreri*. EOs rich in oxygenated components and/or phenylpropanoids may therefore be considered promising candidates for the development of biocontrol agents applicable to agricultural systems.

Based on the IC_50_ values obtained for the phenylpropanoids evaluated, preliminary structure–activity relationships can be proposed in relation to their antifungal activity against *M. roreri.* The data indicate that the presence, number, and position of functional groups on the aromatic ring and the side chain significantly influence inhibitory potency. The most active compounds were myristicin (C29, IC_50_ = 0.48 µL·L^−1^) and dillapiole (C14, IC_50_ = 0.56 µL·L^−1^), both bearing a methylenedioxy-substituted aromatic ring and methoxy groups in ortho and para positions. In contrast, apiole (C3, IC_50_ = 3.66 µL·L^−1^), which also possesses a methylenedioxy group, exhibited approximately sevenfold-lower activity. This difference highlights the importance of the methoxylation pattern, suggesting that the specific positioning of methoxy substituents adjacent to the methylenedioxy group, as observed in apiole, may be less favorable for antifungal efficacy.

The relevance of methoxy groups is further supported by comparison with safrole (C36, IC_50_ = 15.04 µL·L^−1^), which lacks methoxy substituents on the aromatic ring and showed significantly lower activity. In fact, its IC_50_ value was 4 to 30 times higher than that of C3, C14, and C29, reinforcing the idea that methoxy substitution may play a key role in enhancing biological activity. In addition to aromatic substitution, the position of the double bond in the side chain may also contribute to antifungal performance, although to a lesser extent. Comparisons among compounds such as C26 and C27, or C15 and C2 reveal that those with a terminal double bond in the propyl side chain (C15 and C26) were slightly more active than those with conjugation extending into the aromatic ring (C2 and C27). However, the differences in activity between these pairs did not exceed 1.36-fold, indicating that substituents on the aromatic ring exert a stronger influence on activity than modifications to the side chain.

The number of methoxy groups on the ring also appears to play a significant role. Methyl eugenol (C26) and methyl isoeugenol (C27), each containing two methoxy groups, were approximately ten times more active than estragole (C15) and anethole (C2), each of which contains only one. Conversely, α-asarone (C4), with three methoxy groups, exhibited no antifungal activity under the conditions tested. These results suggest that while the introduction of methoxy groups can enhance activity, excessive substitution may lead to a loss of efficacy. Substitution of phenolic hydroxyl groups with methoxy groups was also associated with increased activity. For example, methyl eugenol (C26, IC_50_ = 4.84 µL·L^−1^) was significantly more active than eugenol (C16, IC_50_ = 85.42 µL·L^−1^), representing an 18-fold improvement. A similar trend was observed for isoeugenol and methyl isoeugenol, with only the methylated compound exhibiting inhibition greater than 60% under the experimental conditions. These results suggest that hydroxyl methylation may enhance antifungal activity by altering physicochemical properties such as stability, lipophilicity, and membrane permeability.

Allylbenzene (C1), which lacks oxygenated substituents, displayed lower activity than most oxygenated phenylpropanoids. However, it was more effective than eugenol, isoeugenol, and α-asarone, indicating that the presence of hydroxyl or multiple methoxy groups does not necessarily confer improved efficacy. These findings emphasize that both the presence and the spatial arrangement of functional groups on the aromatic ring are critical for biological performance. Methoxy and methylenedioxy substituents, when appropriately positioned, appear to be particularly favorable for enhancing the antifungal activity of phenylpropanoids against *M. roreri.*

Among cyclic oxygenated monoterpenes, higher antifungal activity was observed when the hydroxyl group was attached to the tertiary carbon of the isopropyl moiety rather than directly to the ring. This trend was evident in the comparison of compounds C38, C18, and C24, with C38 (α-terpineol) showing the greatest activity. The presence of an exocyclic double bond in the side chain also appeared to have a positive influence. Isopulegol (C18), which contains such a feature, was approximately twice as active as menthol (C24), its saturated analog. For cyclic monoterpene ketones, the presence of an α,β-unsaturated carbonyl system did not correlate with increased activity. Dihydrocarvone (C13), which contains a non-conjugated double bond, was more effective than carvone (C9), piperitone (C34), and pulegone (C35). In addition, menthone (C25), lacking double bonds in the ring, was the least active of this group. These results suggest that non-conjugated unsaturations in the carbocyclic skeleton may contribute to antifungal potency.

In the case of acyclic aliphatic ketones, carbon chain length had a notable effect on activity. 2-undecanone (C40), with a longer hydrocarbon chain, was approximately five times more active than 2-nonanone (C31). A similar trend was observed among acyclic alcohols: nerolidol (C30), a sesquiterpenic alcohol with a longer side chain, showed three-fold greater activity than linalool (C23), a monoterpenic counterpart. These observations suggest that, in oxygenated acyclic structures, the length of the hydrocarbon chain may enhance the inhibitory effect of volatile compounds against the pathogen.

Finally, the strong antifungal activity of phenylpropanoids against *M. roreri* underscores their potential as effective biocontrol agents. However, compounds such as safrole, myristicin, and apiole, as well as essential oils rich in phenylpropanoids, have been linked to potential phytotoxic effects in some plant species [70,71,72]. These may include impacts on germination, root growth, or other physiological functions, depending on dose and plant sensitivity. Further research is needed to assess their safety and compatibility with cacao under greenhouse and field conditions to support their use in sustainable disease management.

## 3. Materials and Methods

### 3.1. General Experimental Procedures

GC–MS analyses were performed using a GC 2010 Plus gas chromatograph coupled to a GCMS-TQ 8040 triple quadrupole mass spectrometer (Shimadzu^®^, Kyoto, Japan). The instrument was operated in electron impact mode (70 eV, 100 µA) in full scan acquisition (scan rate: 4.57 s^−1^), covering a mass range of *m*/*z* 40–400. The ion source (trap) and transfer line temperatures were both set at 280 °C. Essential oils (EOs) were analyzed using two capillary columns with orthogonal polarities: a DB-5MS column ((5%)-phenyl-methylpolysiloxane, 60 m × 0.25 mm i.d., 0.25 µm film thickness) and an HP-INNOWax column (polyethylene glycol, 60 m × 0.25 mm i.d., 0.25 µm film) (Agilent Technologies, Santa Clara, CA, USA). Linear retention indices (LRIs) were calculated based on a standard solution of n-alkanes (C7–C40, 1000 ppm; Sigma-Aldrich^®^, St. Louis, MO, USA).

Isolation and purification of natural constituents from the EOs were carried out by flash chromatography (FC) using SiliaFlash^®^ P60 silica gel (particle size 25–40 µm; SiliCycle^®^, Quebec, QC, Canada). Fractions were monitored and analyzed by thin-layer chromatography (TLC) on SiliaPlate™ aluminum sheets coated with silica gel P60 F_254_ (5–20 µm; SiliCycle^®^), using UV light at 254 and 365 nm and iodine vapor for visualization. Solvent removal was conducted using a rotary evaporator (Hei-VAP, Heidolph Instruments GmbH & Co. KG, Schwabach, Germany). All solvents used in chromatographic procedures were of technical grade, commercially acquired, and distilled and dried prior to use. Other reagents were used as received without further purification. Structural characterization of the isolated compounds was performed by ^1^H-NMR and ^13^C-NMR (APT) spectroscopy. NMR spectra were recorded at 400 MHz for ^1^H and 100 MHz for ^13^C using a Bruker Advance AC-400 spectrometer (Bruker^®^, Hamburg, Germany) in CDCl_3_ at 25 °C. Chemical shifts (δ) are reported in parts per million (ppm) and coupling constants (J) in Hertz (Hz).

### 3.2. Screening of Antifungal Activity of Piper EOs Against M. roreri

#### 3.2.1. Plant Material

The samples of 34 plant species of *Piper* genus were randomly collected during different field trips in the departments of Cundinamarca and Boyacá (Colombia). A specimen of each sample gathered was sent to an herbarium (Herbario Nacional Colombiano or Herbario Universidad de Antioquia) for taxonomic determination (Table S1). The collection of plant species was carried out under the contract of access to genetic resources and derived products No. 121 (22 January 2016), with OTROSI No. 21 celebrated between Ministerio de Medio Ambiente y Desarrollo Sostenible and Universidad Nacional de Colombia and under the amnesty framework established in Article 6 of Law 1955 of 2019.

#### 3.2.2. EOs Extraction

Fresh aerial parts of the *Piper* species were subjected to steam distillation for approximately 2 h. The EOs were collected by condensation using a Clevenger-type apparatus, separated by decantation, dried over anhydrous sodium sulfate, and stored at 4 °C in refrigeration until analysis.

#### 3.2.3. Fungal Strain

The *M. roreri* strain used in this study was maintained under controlled conditions at 24 ± 1 °C in the dark and preserved in the fungal collection. Its identity was confirmed through morphological and molecular analyses. Macroscopic and microscopic observations were conducted on colonies grown on potato dextrose agar (PDA) incubated at 25 °C for four weeks. Colony characteristics and reproductive structures were recorded weekly, and microscopic features stained with lactophenol blue were consistent with reference descriptions for *M. roreri* [73,74]. For molecular identification, DNA was extracted from the isolate and the internal transcribed spacer (ITS) region of the ribosomal DNA was amplified using primers ITS4 and ITS5. PCR products were verified on 1% agarose gels and sequenced by AGROSAVIA (Corporación Colombiana de Investigación Agropecuaria, Bogotá-Colombia) using the Sanger method. Sequences were edited with BioEdit (v7.1.9) and compared to GenBank entries using the BLASTn algorithm, showing ≥99% identity and an E-value of 0.0 with *M. roreri* reference sequences.

#### 3.2.4. In Vitro Antifungal Evaluation of the EOs Using a Vapor-Phase Diffusion Assay Against *M. roreri*

The antifungal activity of the essential oils (EOs) was evaluated using an in vitro vapor-phase diffusion assay, a fumigation-type method based on confined vapor exposure, adapted from methods previously reported in the literature [75,76,77,78,79,80]. This approach is particularly suitable for assessing the efficacy of volatile, hydrophobic compounds such as EOs in sealed environments, where conventional contact-based methods are less effective.

The assay was conducted in sterile 90 mm diameter × 15 mm height glass Petri dishes containing potato dextrose agar (PDA) as the culture medium. At the center of each plate, 2 µL of a conidial suspension of *M. roreri* (1 × 10^6^ conidia/mL) was deposited. Surrounding the inoculation site, three sterile Whatman^®^ No. 1 filter paper discs (5 mm in diameter) were affixed equidistantly at approximately 2 cm. A volume of 5 µL of EO was applied to each disc, totaling 15 µL per plate. The Petri dishes were immediately sealed with Parafilm^®^ to prevent vapor loss and to maintain a confined atmosphere. The vapor-phase concentration was expressed in µL/L of air and was calculated based on the total applied volume (15 µL) and the internal headspace of the Petri dish (~82 mL). Under these conditions, the exposure concentration for the initial screening was approximately 183 µL/L of air. Plates were incubated at 24 ± 1 °C in the dark. Fungal growth was monitored daily, and the experiment was terminated once the radial growth in the negative control (EO-free) plates reached approximately 60% of the total plate diameter (~50 mm). This typically occurred between 5 and 7 days post-inoculation. Digital images of each plate were taken, and radial growth was measured using ImageJ version 1.53t. A positive control with Mancozeb^®^ 80 WP (Vecol S.A, Bogotá-Colombia) was included to confirm fungal inhibition (1000 µg/mL). All treatments were performed using three biological replicates, each consisting of four technical replicates. Mycelial growth inhibition (MGI) was calculated using the following formula:

(1) $$\%MGI=\frac{\%MGIc-MGIt}{\%MGIc}\times 100$$

where *MGIc* represents the mycelial growth in the control and *MGIt* corresponds to the growth under treatment.

EOs that showed ≥60% inhibition under screening conditions were considered bioactive and selected for further testing. For these, dose–response assays were performed using volumes ranging from 0.5 µL to 20 µL per dish, corresponding to vapor-phase concentrations between 0.58 and 243 µL/L of air. The median inhibitory concentration (IC_50_) for each EO or pure compound was determined from dose–response data by nonlinear regression analysis (probit model), using IBM SPSS Statistics 28. All results are reported as mean IC_50_ ± standard deviation (SD).

#### 3.2.5. Statistical Analysis

A one-way analysis of variance (ANOVA) was performed using the IC_50_ values as the response variable. Once significant overall differences were detected (*p* < 0.05), a multiple comparison procedure was applied using Tukey’s HSD post hoc test, with the sample size adjusted using the harmonic mean of the groups (n = 4.0).

### 3.3. Chemical Characterization of EOs with Potential Activity Against M. roreri

#### 3.3.1. Sample Preparation

For each sample, 25 μL of EO was diluted with n-hexane to a final volume of 1.0 mL. Similarly, a standard hydrocarbon mixture, (C_7_–C_40_) (Sigma-Aldrich, Saint Louis, MO, USA), was prepared by diluting 25 μL of the stock solution in n-hexane to a final volume of 1.0 mL.

#### 3.3.2. GC–MS Analysis

The chromatographic analyses were performed using two columns. The first was a DB-5MS column, with a 1 μL injection volume and a 20:1 split ratio, at an injection temperature of 280 °C. Helium (99.9995%) was used as the carrier gas at a linear velocity of 25.5 cm/s and a constant flow rate of 1 mL/min. The temperature program started at 40 °C (2 min hold), and then increased to 123 °C at 4 °C/min (2 min hold), followed by a ramp to 160 °C at 4 °C/min (5 min hold), then 220 °C at 5 °C/min (8 min hold), and finally to 280 °C at 5 °C/min (4 min hold), with a total runtime of 75 min. The second analysis used a HP-INNOWax column under the same injection conditions. The temperature program began at 45 °C (4 min hold), ramped to 120 °C at 3 °C/min (2 min hold), and then increased to 250 °C at 4 °C/min (8 min hold), with a total analysis time of 71.5 min.

#### 3.3.3. Tentative Identification of EO Constituents

Chemical compounds were tentatively identified by comparing their mass spectra and linear retention indices (LRIs) with those from the NIST 14.L, Wiley 8.1, and Pherobase databases, along with values reported by Adams [58,81,82]. The LRI values were calculated using a series of n-alkanes run under the same chromatographic conditions as the EO samples [81].

### 3.4. In Vitro Antifungal Evaluation of Selected Chemical Constituents Using a Vapor-Phase Diffusion Assay Against M. roreri

#### 3.4.1. Chemicals

The plant-derived volatile compounds (VCs) evaluated in this study were selected based on two criteria: (i) their presence in essential oils that showed antifungal activity against *M. roreri*, and (ii) their structural similarity to those constituents, allowing for a structure–activity relationship (SAR) analysis. Of the 40 compounds tested, 35 were commercially sourced, as detailed in Supplementary Materials’ Table S2. Estragole, apiole, dillapiole and myristicin had been previously isolated from the essential oils of *Artemisia dracunculus*, *P. holtonii*, *P. aduncum*, and *P. asperiusculum*, and were available in the laboratory (Table S2) [83,84,85]. Piperitone was isolated via flash chromatography from the EOs of *P. asperiusculum* and was characterized by NMR and GC-MS (Tables S4 and S5, Figures S2–S4).

#### 3.4.2. In Vitro Antifungal Evaluation of the VCs Using a Vapor-Phase Diffusion Assay

The antifungal activity of the volatile compounds (VCs) was evaluated using the same vapor-phase methodology described for the EOs in Section 3.2.4, using a maximum concentration of 122 μL/L air. Compounds that exhibited ≥ 60% mycelial growth inhibition were considered active and were further evaluated to determine their median inhibitory concentration (IC_50_). IC_50_ values were estimated by nonlinear regression analysis using a concentration range between 0.48 and 122 μL/L air. All assays were conducted with four replicates, and standard deviations were calculated. The data are presented as mean IC_50_ ± standard deviation (SD).

#### 3.4.3. Statistical Analysis

A one-way analysis of variance (ANOVA) was performed using the IC_50_ values as the response variable. Once significant overall differences were detected (*p* < 0.05), a multiple comparison procedure was applied using Tukey’s HSD post hoc test, with the sample size adjusted using the harmonic mean of the groups (n = 4.0).

## 4. Conclusions

This study provides new evidence of the antifungal effectiveness of *Piper*-derived essential oils and volatile compounds in the vapor phase against *M. roreri*. For the majority of the tested essential oils and all individual constituents, this constitutes the first report of activity against this phytopathogen. Notably, several oils showed high efficacy, particularly those rich in phenylpropanoids and oxygenated compounds. The EOs of *Piper holtonii* and *Piper aduncum* were the most active, and among the individual compounds, myristicin and dillapiole showed the highest antifungal activity. The preliminary structure–activity relationship (SAR) analysis revealed structural features associated with antifungal potency. In phenylpropanoids, increased activity was linked to the presence of methoxy substituents and methylenedioxy groups on the aromatic ring, as well as to the methylation of phenolic hydroxyl groups. Among cyclic monoterpenoids, the most active compounds were those containing oxygenated functionalities such as alcohols and ketones, particularly when the structure also included non-conjugated double bonds. In acyclic compounds, including aliphatic ketones and alcohols, a longer hydrocarbon chain was associated with greater antifungal efficacy. Overall, these findings highlight the potential of *Piper* species as a valuable source of antifungal volatiles and support their use in the development of natural products for the sustainable control of *M. roreri* in cacao cultivation.

## Figures and Tables

**Table 1:** Inhibitory concentrations (IC_50_) of EOs from bioactive *Piper* species against *M. roreri*.

N°	Specie	IC_50_ (µL/L air) ± SD	Subsets *
A18	*P. holtoni*	0.58 ± 0.07	A
A2	*P. aduncum*	0.61 ± 0.16	A
A1	*P. aduncum*	0.62 ± 0.08	A
A29	*P statarium*	2.05 ± 0.15	A
A11	*P. divortans*	12.54 ± 0.11	B
A7	*P. auritum*	15.03 ± 0.85	B
A34	*P. tuberculatum*	22.71 ± 0.17	C
A10	*P. cumbricola*	35.45 ± 0.54	D
A30	*P. subflavum*	41.57 ± 0.62	E
A5	*P. asperísculum*	44.88 ± 2.04	E
A14	*P. eriopodon*	54.76 ± 1.54	F
A23	*P. marginatum* var. *niceforoi*	60.14 ± 0.80	G
A21	*P. marginatum*	61.87 ± 1.09	G
A31	*P. tenue*	82.73 ± 3.51	H
A17	*P. haughtii*	85.61 ± 1.41	H-I
A12	*P. elbancoanum*	89.17 ± 0.22	I-J
A3	*P. aequale*	90.20 ± 2.75	J
A4	*P. albomaculatum*	125.79 ± 0.86	K
A13	*P. elmetanum*	150.13 ± 2.94	L
A20	*P. lanceifolium*	184.27 ± 1.09	M

The data presented are expressed as the mean of four replicates (n = 4) and three repetitions with the respective confidence intervals, with *p* ≤ 0.05 for the nonlinear model—probit analysis. The lethal concentrations were obtained with a 95% confidence limit. * Post hoc Tukey’s multiple comparison analysis (Supplementary Materials, Figure S1).

**Table 2:** Major volatile components (% area) identified by GC-MS in bioactive EOs from *Piper* species.

N°	Essential Oil	Majority Compounds (%)
A1	*P. aduncum*	α-copaene (3.3%), caryophyllene (6.3%), germacrene D (7.0%), myristicin (22.9%), germacrene B (4.2%), dillapiole (29.5%).
A2	*P. aduncum*	Z-β-ocimene (3.7%), α-copaene (5.5%), caryophyllene (7.6%), germacrene D (7.0%), myristicin (5.6%), germacrene B (7.2%), viridiflorol (4.1%), dillapiole (23.3%), apiole (7.3%).
A3	*P. aequale*	β-pinene (3.1%), limonene (9.7%), safrole (3.8%), α-copaene (3.4%), β-elemene (4.4%), β -caryophyllene (10.8%), germacrene D (8.9%), germacrene B (11.0%), E-nerolidol (11.8%), spathulenol (4.2%).
A4	*P. albomaculatum*	γ-terpinene (3.8%), linalool (7.1%), α-copaene (6.4%), β-elemene (4.7%), caryophyllene (8.6%), alloaromadendrene (3.1%), selina-5,11-diene (6.9%), humulene (3.9%), germacrene D (5.6%), cadina-1(6),4-diene (5.1%), germacrene B (4.7%).
A5	*P. asperiusculum*	α-pinene (3.8%), α-phellandrene (4.4%), limonene (7.9%), piperitone (69.8%), isocaryophyllene (4.9%).
A7	*P. auritum*	γ-terpinene (5.1%), α-terpinolene (6.3%), camphor (10.1%), safrole (64.3%).
A10	*P. cumbricola*	p-cymene (3.0%), limonene (5.6%), γ-terpinene (3.8%), linalool (4.0%), E-p-menth-2-en-1-ol (4.4%), z-p-mentha-2-en-1-ol (4.2%), terpinen-4-ol (15.8%), piperitone (5.0%), isocaryophyllene (4.5%).
A11	*P. divortans*	sabinene (3.1%), limonene (3.0%), e-β-ocimene (3.0%), γ-terpinene (3.6%), piperitone (7.5%), α-copaene (4.3%), caryophyllene (8.2%), humulene (3.8%), germacrene D (5.1%), cadina-1(6),4-diene (4.7%), germacrene B (4.6%), viridiflorol (3.1%), dillapiole (7.3%), apiole (6.9%).
A12	*P. elbancoanum*	sabinene (3.2%), β-myrcene (3.3%), limonene (41.8%), safrole (6.1%), E-nerolidol (6.5%), spathulenol (4.6%), guaiol (7.6%).
A13	*P. elmetanum*	α-pinene (7.4%), o-cymene (17.6%), limonene (18.3%), cryptone (3.2%), geraniol (5.8%), 2-hydroxy-1,8-cineole (9.1%), caryophyllene oxide (4.4%).
A14	*P. eriopodon*	α-pinene (11.6%), β-pinene (11.4%), α-copaene (8.9%), caryophyllene (13.8%), alloaromadendrene (4.0%), cadina-1(6),4-diene (3.7%), eudesma-4(14),11-diene (5.0%), germacrene B (4.5%), apiole (3.8%).
A17	*P. haughtii*	γ-terpinene (5.7%), α-copaene (11.5%), germacrene D (14.0%), cadina-1(6),4-diene (6.3%), caryophyllene (12.6%), germacrene B (3.8%), E-nerolidol (4.4%), caryophyllene oxide (3.0%), apiole (3.0%).
A18	*P. holtoni*	α-pinene (3.1%), β-pinene (3.0%), caryophyllene (5.8%), germacrene D (11.0%), germacrene B (3.9%), spathulenol (3.0%), dillapiole (22.0%), apiole (37.2%)
A20	*P. lanceifolium*	α-cubebene (3.5%), α-copaene (3.8%), caryophyllene (24.0%), germacrene B (4.2%), eudesm-4(14)-en-11-ol (10.7%), caryophyllene oxide (6.1%), γ-eudesmol (4.8%), farnesyl alcohol (14.7%), cembrene (3.5%).
A22	*P. marginatum*	estragole (60.9%), E-Anethole (3.8%), α-copaene (4.5%), β-elemene (3.2%), caryophyllene (6.0%), germacrene B (3.1%).
A23	*P. marginatum* var. *niceforoi*	γ-terpinene (4.4%), estragole (57.6%), E-Anethole (7.8%), α-copaene (5.2).
A29	*P. statarium*	α-thujene (3.8%), α-pinene (5.0%), β-pinene (5.8%), α-copaene (4.2%), germacrene D (3.8%), myristicin (64.2%).
A30	*P. subflavum*	α-pinene (16.9%), β-pinene (18.5%), limonene (4.9%), γ-terpinene (4.3%), α-copaene (8.2%), caryophyllene (7.3%), germacrene D (8.4%), germacrene B (3.7%), apiole (3.4%)
A31	*P. tenue*	α-pinene (4.3%), β-pinene (4.4%), 2-carene (4.3%), α-phellandrene (3.5%), p-cymene (3.5%), limonene (6,4%), Z-β-ocimene (6.2%), E-β-ocimene (3.5%), γ-terpinene (6.3%), α-terpinolene (3.6%), terpinen-4-ol (8.6%), caryophyllene (5.7%).
A34	*P. tuberculatum*	α-pinene (3.3%), β-pinene (4.2%), Z-β-ocimene (4.4%), α-copaene (3.8%), caryophyllene (9.9%), germacrene D (10.8%), myristicin (9.2%), germacrene B (4.3%), E-nerolidol (5.4%), apiole (13.9%).

**Table 3:** IC_50_ values of volatile bioactive compounds against *M. roreri*.

Compound	*Structure*	IC_50_ ± SD	Subsets *	Compound	*Structure*	IC_50_ ± SD	Subsets *
Myristicin (C28)	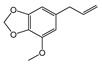	0.48 ± 0.05	A	Piperitone (C34)	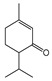	28.06 ± 4.16	F
Dillapiole (C14)	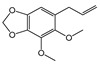	0.56 ± 0.08	A	Estragole (C15)	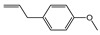	41.48 ± 0.85	G
Apiole (C3)	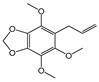	3.66 ± 0.51	A-B	trans-Anethole (C2)	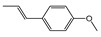	42.16 ± 1.27	G
Citral (C11)	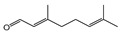	3.99 ± 0.41	A-B	Terpinolene (C39)	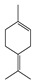	42.70 ± 1.45	G
(S)-(-)-α-Terpineol (C38)	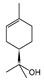	4.27± 2.56	A-B	(−)-Menthol (C23)	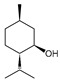	45.18 ± 0.15	G
Methyl eugenol (C25)	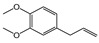	4.80 ± 2.47	A-B	Allylbenzene (C1)	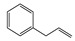	59.65 ± 1.26	H
Methyl isoeugenol (C26)	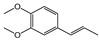	6.59 ± 3.33	A-B-C	2-Nonanone (C30)	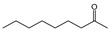	60.24 ± 0.51	H
E-Cinnamaldehyde (C10)	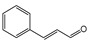	7.26 ± 4.41	A-B-C	(−)-Linalool (C22)	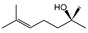	73.20 ± 3.33	I
2-Undecanone (C40)	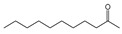	12.25 ± 1.26	B-C-D	R-(+)-Pulegone (C35)	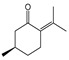	75.64 ± 3.33	I
Safrole (C36)	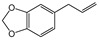	15.04 ± 1.48	C-D-E	Eugenol (C16)	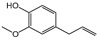	85.42 ± 1.48	J
Dihydrocarvone (C13)	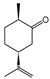	17.45 ± 4.12	D-E	Menthone (C24)	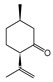	85.47 ± 0.78	J
E-Nerolidol (C29)	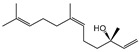	21.96 ± 2.78	E-F	R-(-)-Fenchone (C17)	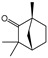	89.43 ± 1.07	J
R-(-)-Carvone (C9)	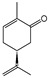	22.87 ± 0.41	E-F	γ-Terpinene (C37)	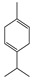	103.70 ± 7.24	K
(−)-Isopulegol (C18)	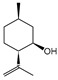	22.45 ± 0.33	E-F	R-(-)-α-Phellandrene (C31)	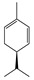	109.81 ± 6.47	K

The data presented are expressed as the mean of three replicates (n = 3) and three repetitions with the respective confidence intervals, with *p* ≤ 0.05 for the nonlinear model - probit analysis. The lethal concentrations obtained with a 95% confidence limit. * Post hoc Tukey multiple comparisons analysis (Supplementary Data, annex 6, Figure S5).

## Data Availability

Data are contained within this article and the Supplementary Materials.

## Supplementary Materials

The following supporting information can be downloaded at: https://www.mdpi.com/article/10.3390/plants14162514/s1. Table S1: Collection information of the 34 *Piper* species evaluated; Table S2: Selected volatile chemical constituents for fumigant evaluation against *M. roreri*; Table S3: Chemical compositions of the EOs of 20 essential oils from *Piper* species with potential fungicidal activity; Figure S1: Table of results of the Tukey test EOs; Table S4: Spectroscopic data and NMR spectra of compound C34 [86]; Figure S2: NMR ^1^H spectrum C34 (CDCl_3_, 400 MHz); Figure S3: NMR APT spectrum (CDCl3, 100 MHz); Figure S4: Chromatographic profile (TIC) from GC-MS analysis (DB-5MS) of piperitone isolated from *Piper asperisculum*; Table S5: Chemical composition of piperitone isolated from *Piper asperisculum* (DB-5MS); Figure S5: Table of results of the compound Tukey test.

## Abbreviations

The following abbreviations are used in this manuscript:

ANOVA	A one-way analysis of variance
APT	Attached Proton Test
CDCl_3_	Deuterated chloroform
^13^C-NMR	Carbon Nuclear Magnetic Resonance
d	Doublet
DCM	Dichloromethane
EI	Electron impact
EO	Essential oil
EOs	Essential oils
eV	Electron volt
ExpLRI	Experimental Linear Retention Index
FC	Flash chromatography
g	Grams
CG	Gas chromatography
GC–MS	Gas chromatography with mass spectrometry
GCMS-TQ	Gas chromatography–mass spectrometry with triple quadrupole
Hz	Hertz
^1^H-NMR	Proton Nuclear Magnetic Resonance
IC_50_	Median inhibitory concentration
i.d	Internal diameter
J	Coupling constant
L	Liter
m	Multiplet
m	Meters
mg	Milligrams
MGI	Mycelial growth inhibition
MHz	Megahertz
min	Minutes
mL	Milliliter
mm	Millimeter
*m*/*z*	Mass/charge
nm	Nanometers
NMR	Nuclear Magnetic Resonance
*p*	*p* value
ppm	Parts per million
q	Quartet
rt	Retention time
Ref	Reference
REF LRI	Reference linear retention index
s	Singlet
SD	Standard deviation
UV	Ultraviolet
VCs	Volatile compounds
°C	Degree Celsius
δ	Chemical shift
δC	Carbon shift
δH	Hydrogen shift
µL	Microliter
µm	Micrometer
