# Supplementary material for: Antifungal Potential of Piper-Derived Essential Oils and Key Constituents on Moniliophthora roreri, the Causal Agent of Moniliasis in Cacao (Theobroma cacao L.)
Source: Plants (Basel). 2025 Aug 13;14(16):2514. doi: 10.3390/plants14162514 (PMC12389601; doi:10.3390/plants14162514)
Supplement: Supplementary file 1 [file plants-14-02514-s001.zip › plants-3714408-supplementary.pdf]

Supplementary Material

1. Plant material. Characterization of the aromatic species used according to origin, plant organ, and regulatory support.

Table S1. Collection information of the 34 *Piper* species evaluated.

N°	Specie	Plant part used	collection site	Herbarium code	Permit
A1	*Piper aduncum L.*	Aerial Part	San Antonio del Tequendama	COL631636	CARG
A2	*Piper aduncum L.*	Aerial Part	Otanche (Boyacá)	COL631624	CARG
A3	*Piper aequale Vahl*	Aerial Part	Pauna (Boyacá)	COL631612	CARG
A4	*Piper albomaculatum D.Dietr.*	Aerial Part	Otanche (Boyacá)	COL631623	CARG
A5	*Piper arboreum Aubl.*	Aerial Part	Otanche (Boyacá)	Determined but not deposited in a herbarium	Amnesty
A6	*Piper asperiusculum Kunth.*	Aerial Part	San Mateo (Boyacá)	COL 579924	Amnesty
A7	*Piper auritum Kunth*	Aerial Part	Otanche (Boyacá)	HUA 217580	Amnesty
A8	*Piper bredemeyeri J.Jacq.*	Aerial Part	Otanche (Boyacá)	COL631616	CARG
A9	*Piper cumanense Kunth*	Aerial Part	El Colegio (Cundinamarca)	COL631641	CARG
A10	*Piper cumbricola Trel*	Aerial Part	Santa Bárbara (Santander)	HUA 217590	Amnesty
A11	*Piper divortans Trel. & Yunck.*	Aerial Part	Pauna (Boyacá)	COL631613	CARG
A12	*Piper elbancoanum Trel. & Yunck.*	Aerial Part	Otanche (Boyacá)	COL631627	CARG
A13	*Piper elmetanum Trel. & Yunck.*	Aerial Part	Acacias (Meta)	COL553310	Amnesty
A14	*Piper eriopodon (Miq.) C.DC.*	Aerial Part	El Colegio (Cundinamarca)	COL631642	CARG
A15	*Piper falcifolium Trel.*	Aerial Part	Otanche (Boyacá)	COL631622	CARG
A16	*Piper grande Vahl.*	Aerial Part	Puerto Boyacá (Boyacá)	HUA 217596	Amnesty
A17	*Piper haughtii Trel. &*	Aerial Part	Otanche	COL631621	CARG

	Yunck.		(Boyacá)		
A18	*Piper holtonii C.DC.*	Aerial Part	El Colegio (Cundinamarca)	COL593726	Amnesty
A19	*Piper imperiale (Miq.) C.DC.*	Aerial Part	Duitama (Boyacá)	COL519826	Amnesty
A20	*Piper lanceifolium Kunth.*	Aerial Part	Otanche (Boyacá)	HUA 217605	Amnesty
A21	*Piper marequitense C.DC.*	Aerial Part	San Antonio del Tequendama (Cundinamarca)	COL631633	CARG
A22	*Piper marginatum Jacq.*	Aerial Part	Otanche (Boyacá)	COL631614	CARG
A23	*P marginatum var.niceforoi (trel. & Yunck.) Steyerm*	Aerial Part	Otanche (Boyacá)	COL631629	CARG
A24	*Piper pertomentellum Trel. & Yunck.*	Aerial Part	San Mateo (Boyacá)	COL579920	CARG
A25	*Piper reticulatum L.*	Aerial Part	Otanche (Boyacá)	COL631628	CARG
A26	*Piper rusticum Trel. & Yunck.*	Aerial Part	El Colegio (Cundinamarca)	COL631640	CARG
A27	*Piper sp.*	Aerial Part	San Antonio del Tequendama (Cundinamarca)	COL631625	CARG
A28	*Piper sp.*	Aerial Part	Otanche (Boyacá)	COL631618	CARG
A29	*Piper statarium Trel. & Yunck.*	Aerial Part	Otanche (Boyacá)	HUA 217602	Amnesty
A30	*Piper subflavum var. espejuelanum C.DC.Trel. & Yunck.*	Aerial Part	San Antonio del Tequendama (Cundinamarca)	COL631631	CARG
A31	*Piper tenue Kunth.*	Aerial Part	Otanche (Boyacá)	HUA 217594	Amnesty
A32	*Piper tomas-albertoi Trel. & Yunck.*	Aerial Part	San Antonio del Tequendama (Cundinamarca)	COL631632	CARG
A33	*Piper trachydermum Trel.*	Aerial Part	Otanche (Boyacá)	COL631619	CARG
A34	*Piper tuberculatum Jacq.*	Aerial Part	El Colegio (Cundinamarca)	COL631638	CARG

2. Sample information of the 40 chemical constituents of essential oils worked.

Table S2. Selected volatile chemical constituents for fumigant evaluation against *M. roreri*.

N°	Compound	Structure	Purity (%)	Compound type	Source
**C1**	Allylbenzene	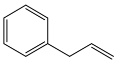	98.0	Phenylpropanoid	Tokio Chemical Industry ® Tokio, Japan
**C2**	trans-Anethole	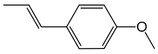	98.0	Phenylpropanoid	Merck ®, Darmstadt, Germany.
**C3**	Apiole	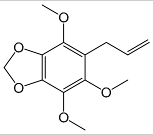	>90.0*	Phenylpropanoid	Insoled of *P. holtoni* [84]
**C4**	α -Asarone	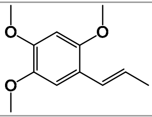	99.0	Phenylpropanoid	Angene, Nanjing, China
**C5**	(+)-2-Bornanone	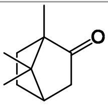	97.0	Oxygenated monoterpenoid	Sigma Aldrich® Saint Louis, MO, USA
**C6**	δ-3-Carene	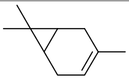	90.0	Hydrocarbon-type monoterpenoid	Sigma Aldrich® Saint Louis, MO, USA
**C7**	(+)-4-Carene	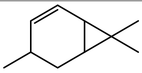	>90.0	Hydrocarbon-type monoterpenoid	Sigma Aldrich® Saint Louis, MO, USA
**C8**	β-Caryophyllene	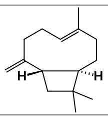	80.0	Hydrocarbon-type sesquiterpenoid	Sigma Aldrich® Saint Louis, MO, USA
**C9**	R-(-)-Carvone	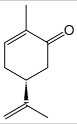	98.0	Oxygenated monoterpenoid	Sigma Aldrich® Saint Louis, MO, USA
**C10**	E- Cinnamaldehyde	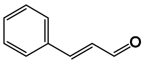	98.0	Phenylpropanoid	Merck ®, Darmstadt, Germany.
**C11**	Citral	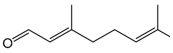	95.0	Oxygenated monoterpenoid	Sigma Aldrich® Saint Louis, MO, USA
**C12**	1,8-Cineole	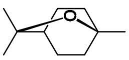	99.0	Oxygenated monoterpenoid	Sigma Aldrich® Saint Louis, MO, USA
**C13**	(+)-Dihydrocarvone	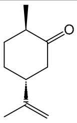	>90%	Oxygenated monoterpenoid	Sigma Aldrich® Saint Louis, MO, USA

**C14**	Dillapiole	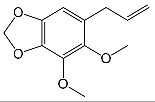	>90.0*	Phenylpropanoid	Insoled of *P. aduncum* [84]
**C15**	Estragole	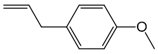	95.0	Phenylpropanoid	Insoled of A. *dracunculus* [85]
**C16**	Eugenol	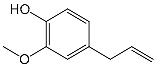	98.0	Phenylpropanoid	Sigma Aldrich® Saint Louis, MO, USA
**C17**	R-(-)-α-Phellandrene	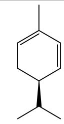	95.0	Hydrocarbon-type monoterpenoid	Sigma Aldrich® Saint Louis, MO, USA
**C18**	R-(-)-Fenchone	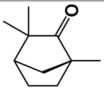	98.0	Oxygenated monoterpenoid	Sigma Aldrich® Saint Louis, MO, USA
**C19**	(-)-Isopulegol	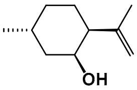	98.0	Oxygenated monoterpenoid	Sigma Aldrich® Saint Louis, MO, USA
**C20**	Isoeugenol	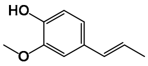	98.0	Phenylpropanoid	Sigma Aldrich® Saint Louis, MO, USA
**C21**	DL-Limonene (1:1)	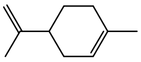	95.0	Hydrocarbon-type monoterpenoid	Merck ®, Darmstadt, Germany.
**C22**	Limonene epóxide	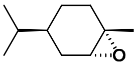	97.0	Oxygenated monoterpenoid	Sigma Aldrich® Saint Louis, MO, USA
**C23**	(-)-Linalool	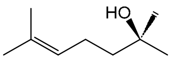	97.0	Oxygenated monoterpenoid	Merck ®, Darmstadt, Germany.
**C24**	(-)-Menthol	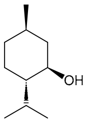	>90.0	Oxygenated monoterpenoid	Sigma Aldrich® Saint Louis, MO, USA
**C25**	Menthone	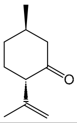	90.0	Oxygenated monoterpenoid	Sigma Aldrich® Saint Louis, MO, USA
**C26**	Methyleugenol	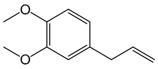	95.0	Phenylpropanoid	Angene, Nanjing, China
**C27**	Methylisoeugenol	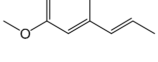	98.0	Phenylpropanoid	Sigma Aldrich® Saint Louis, MO, USA
**C28**	β-Myrcene	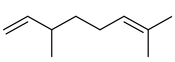	80.0	Hydrocarbon-type monoterpenoid	Aaron chemicals LLC ® California, USA
**C29**	Myristicin	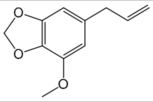	>90.0*	Phenylpropanoid	Insoled of *P. asperísculum* [84]
**C30**	E-Nerolidol	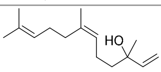	98.0	Oxygenated sesquiterpenoid	Merck ®, Darmstadt, Germany..

**C31**	2-Nonanone	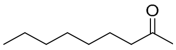	99.0	Aliphatic ketone	Sigma Aldrich® Saint Louis, MO, USA
**C32**	α-Pinene	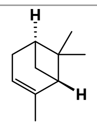	97.0	Hydrocarbon-type monoterpenoid	Merck ®, Darmstadt, Germany..
**C33**	β-Pinene	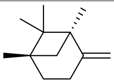	98.0	Hydrocarbon-type monoterpenoid	Merck ®, Darmstadt, Germany..
**C34**	Piperitone	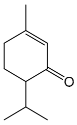	94.9*	Oxygenated monoterpenoid	Insoled of *P. asperísculum (Annex 5, C34)*
**C35**	R-(+)-Pulegone	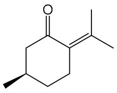	90.0	Oxygenated monoterpenoid	Sigma Aldrich® Saint Louis, MO, USA
**C36**	Safrole	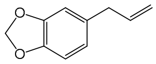	97.0	Phenylpropanoid	Sigma Aldrich® Saint Louis, MO, USA
**C37**	γ-Terpinene	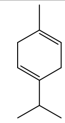	97.0	Hydrocarbon-type monoterpenoid	Sigma Aldrich® Saint Louis, MO, USA
**C38**	(S)-(-)-α-Terpineol	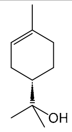	96.0	Oxygenated monoterpenoid	Alpha Aesar ®, Heysham, UK
**C39**	Terpinolene	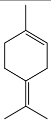	85.0	Hydrocarbon-type monoterpenoid	Sigma Aldrich® Saint Louis, MO, USA
**C40**	2-Undecanone	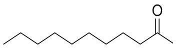	98.0	Aliphatic ketone	Sigma Aldrich® Saint Louis, MO, USA
*	Purity determined by GC-MS				

3. Chemical composition of essential oils from *Piper*.

Table S3. Chemical composition of the 20 essential oils from *Piper* species with potential fungicidal activity.

		DB-5MS		HP-INNOWax		*P. aduncum* (A2)	*P. aduncum* (A1)	*P. aequale* (A3)	*P. albomaculatum* (A4)	*P. asperisculum* (A5)	*P. auritum* (A7)	*P. cumbricola* (A10)	*P. divortans* (A11)	*P. elbancoanum* (A12)	*P.elmetanum* (A13)	*P.eriopodon* (A14)	*P. haughtii* (A17)	*P. holtoni* (A18)	*P. lanceifolium* (A20)	*P marginatum* (A22)	*P, marginatum* (A23)	*P statarium* (A29)	*P. subflavum* (A30)	*P. tenue* (A31)	*P. tuberculatum* (A34)
**N°**	**Components**	**IRE**	**IRT**	**IRE**	**IRT**																				
1	α-Thujene	930	923-931	1005	1012-1039	-	-			-	-	-	-	-	0.7	-	-	-	-	-	-	-	-	3.8	-
2	α-Pinene	932	910-944	981	1000-1040		1.4	2.8	0.7	3.8	0.5	0.6	2.4	1.1	7.4	11.6	1.0	3.1	-	0.6	1.2	5.0	16.9	4.3	3.3
3	Camphene	952	929-968	1064	1040-1083	-	-	-	-	-	0.8	-	-	-	-	-	-		-	-	-	-	-	-	-
4	Sabinene	975	973-976	1170	1123-1147	-	-	-	-	-	-	-	3.1	3.2	1.8	-	0.6	-	-	-	-	1.2	-	-	1.6
5	β-Pinene	980	960-990	1095	1096-1120	0.5	2.6	3.1	0.5	0.5	0.8	0.8	1.8	0.9	0.5	11.4	1.7	3.0	-	0.7	1.1	5.8	18.5	4.4	4.2
6	β-Myrcene	993	969-993	1131	1724-1748	-	-	-	-	0.8	0.7	0.7	0.9	3.3	1.0	0.6	-	-	-	-	-	0.6	1.8	2.3	-
7	2-Carene	1001	1004-1011			-	-	0.8	1.6	-	1.0	-	1.5	-	-	-	2.3	-	0.8	0.6	2.4	-	1.7	4.3	-
8	4-Carene	1003	1004-1014			-	-	-	-	-	-	2.1	-	-	-	-	-	-	-	-	-	-	-	-	-
9	α-Phellandrene	1004	985-1011	1170	1157-1176	-	-	0.7	-	4.4	-	2.5	1.7	-	-	-	-	-	-	-	-	-	0.7	3.5	-
10	3-Carene	1010	1004-1011	1140	1142-1154	-	-	-	-	-	1.5	-	-	-	-	-	-	-	-	-	-	-	-	-	-
11	2-p-Menthen-1-ol	1016	1116-1137			-	-	-	-	-	-	-	-	-	-	-	-	-	-	-	-	-	-	0.6	-
12	o-Cymene	1020	1008-1032			-	-	-	-	0.7	-	-	-	-	17.6	-	-	-	-	-	-	-	-	-	-
13	p-Cymene	1025	1008-1032	1238	1261-1290	-	-	-	2.3	-	-	3.0	0.8	-	-	0.7	2.5	-	-	-	-	-	-	3.5	-
14	Limonene	1030	1023-1038	1195	1198-1234	-	-	9.7	0.9	7.9	1.7	5.6	3.0	41.8	18.3	1.5	2.6	-	-	1.5	-	1.1	4.9	6.4	0.5
15	Eucalyptol	1032	1033-1039	1173	1186-1230	-	-	-	-	-	-	-	-	-	-	-	-	-	-	-	-	-	1.2	-	-
16	z-β-Ocimene	1039	1023-1050	1215	1242-1252	-	3.7	-	-	-	2.7	2.4	-	-	-	1.5	-	-	-	-	0.8	-	-	6.2	4.4
17	β-Phellandrene	1047	1007-1062			-	2.3	-	-	-	-	-	-	-	-	-	-	-	-	-	-	-	-	-	-
18	E-β-Ocimene	1049	1039-1061	1210	1242-1261	1.1	1.5	-	-	-	2.3	0.9	3.0	-	-	0.6	0.6	-	-	-	0.5	0.6	-	3.5	0.8
19	γ-Terpinene	1061	1060-1064	1242	1221-1262	-	-	1.5	3.8	-	5.1	3.8	3.6	0.7	-	-	5.7	-	1.5	1.2	4.4	-	4.3	6.3	-
20	Isoterpinolene	1085	1086-1090			-	-	-	2.3	-	-	-	2.3	-	-	-	-	-	-	-	-	-	0.6	-	-
21	α-Terpinolene	1087	1079-1089	1260	1275-1297	-	1.2	0.9	-	-	6.3	2.8	-	1.0	-	-	2.3	-	-	0.9	2.7	-	-	3.6	-
22	Sabinene hydrate	1096	1068-1097	1507	1450-1469	-	-	-	-	-	-	0.7	-	-	0.6	-	-	-	-	-	-	-	-	0.5	-
23	Linalool	1097	1098-1112	1519	1506-1537	0.6	0.7	-	7.1	0.9	-	4.0	-	-	-	-	1.0	-	-	-	-	-	1.6	-	-
24	Butanoic acid, 2-methyl-, 3-methylbutyl ester	1109	1107-1110			-	-	-	-	-	-	-	-	-	-	-	-	-	-	-	-	-	-	0.7	-

25	E-p-Menth-2-en-1-ol	1115	1116-1137	1533	1542-1608	-	-	-	-	-	-	4.4	-	-	-	-	-	-	-	-	-	-	-	1.0	-
26	(Z)-p-Mentha-2-en-1-ol	1123	1106-1128			-	-	-	-	-	-	4.2	-	-	-	-	-	-	-	-	-	-	-	-	-
27	Neoalloocimene	1130	1128-1132			-	-	-	-	-	-	-	-	-	-	-	-	-	-	-	-	-	-	0.9	-
28	Camphor	1142	1141-1144	1514	1509-1529	-	-	-	-	-	10.1	-	-	-	-	-	1.2	-	-	-	-	-	-	-	-
29	E-p-Menth-1-en-3-ol	1147	1120-1147			-	-	-	-	-	-	2.8	-	-	-	-	-	-	-	-	-	-	-	-	-
30	Menthone	1153	1150-1154	1432	1440-1492	-	-	-	-	-	-	-	-	-	-	0.9	-	-	-	-	-	-	-	-	-
31	endo-Borneol	1167	1150-1169	1665	1653-1721	-	-	-	-	-	-	0.7	-	-	-	-	-	-	-	-	-	-	-	-	-
32	Terpinen-4-ol	1176	1177-1179			-	-	-	-	-	-	15.8	2.2	-	-	-	-	-	-	-	-	-	-	8.6	-
33	Cryptone	1184	1183-1188	1676	1645-1713	-	-	-	-	-	-	-	-	-	3.2	-	-	-	-	-	-	-	-	-	-
34	m-Cymen-8-ol	1185	1183-1188			-	-	-	-	-	-	-	-	-	2.3	-	-	-	-	-	-	-	-	-	-
35	p-Cymen-8-ol	1188	1183-1188	1855	1820-1864	-	-	-	-	-	-	-	-	-	0.5	-	-	-	-	-	-	-	-	-	-
36	Estragole	1195	1158-1206	1525	1506-1537	-	-	-	0.6	-	-	-	-	1.4	-	-	-	-	-	60.9	57.6	-	-	-	-
37	E-Dihydrocarvone	1195	1193-1218			-	-	-	-	-	-	-	-	-	-	0.8	-	-	-	-	-	-	-	-	-
38	α-Terpineol	1198	1185-1207			-	-	-	-	-	-	2.7	-	-	-	-	-	-	-	-	-	-	0.7	0.6	-
39	Cosmen-2-ol	1207	1208-1208			-	-	-	-	-	-	-	-	-	0.8	-	-	-	-	-	-	-	-	-	-
40	Geraniol	1212	1221-1277			-	-	-	-	-	-	-	-	-	5.8	-	-	-	-	-	-	-	-	-	-
41	Z-Carveol	1220	1188-1222			-	-	-	-	-	-	-	-	-	0.7	-	-	-	-	-	-	-	-	-	-
42	2-Hydroxy-1,8-cineole	1232	1229-1240			-	-	-	-	-	-	-	-	-	9.1	-	-	-	-	-	-	-	-	-	-
43	Pulegone	1240	1237-1244			-	-	-	-	-	-	-	-	-	-	0.8	-	-	-	-	-	-	-	-	-
44	Z-2-Decenal	1250	1249-1254			-	-	-	-	-	-	-	-	-	-	1.3	-	-	-	-	-	-	-	-	-
45	Carvone	1252	1242-1253			-	-	-	-	-	-	-	-	-	-	1.4	-	-	-	-	-	-	-	-	-
46	Piperitone	1254	1250-1256	1738	1743-1748	-	-	-	1.4	69.8	-	5.0	7.5	-	-	-	1.3	-	-	-	-	-	-	1.9	-
47	Linalyl acetate	1256	1248-1257	1535	1731-1778	-	-	-	-	-	-	-	-	-	0.5	-	-	-	-	-	-	-	-	-	-
48	E- Asacaridolicol	1275	1270-1273			-	-	-	-	-	-	-	-	-	1.7	-	-	-	-	-	-	-	-	-	-
49	E-Anethol	1283	1285-1289	1830	1809-1847	-	-	-	-	-	-	-	-	-	-	-	-	-	-	3.8	7.8	-	-	-	-
50	Bornyl acetate	1290	1270-1296	1566	1567-1600	-	-	-	-	-	-	-	-	-	0.5	-	-	-	-	-	-	-	-	-	-
51	Safrole	1293	1262-1287	1904	1898-1903	-	-	3.8	-	-	64.3	-	-	6.1	0.5	0.7	-	-	-	1.3	2.5	-	-	-	-
52	Isoascaridole	1302	1269-1305			-	-	-	-	-	-	-	-	-	2.4	-	-	-	-	-	-	-	-	-	-
53	Myrtenyl acetate	1323	1322-1325			-	-	-	-	-	-	-	-	-	-	0.6	-	-	-	-	-	-	-	-	-
54	α-Terpinyl acetate	1340	1349-1351			-	-	-	-	-	-	-	-	0.7	-	-	-	-	-	-	-	-	-	-	-
55	Limonene-1,2-diol	1343	1321-1343			-	-	-	-	-	-	-	-	-	1.9	-	-	-	-	-	-	-	-	-	-
56	α-Cubebene	1352	1342-1396			-	-	-	1.6	-	-	-	-	-	-	-	1.5	-	3.5	-	-	-	-	-	0.5
57	α-copaene	1376	1372-1392	1496	1473-1535	3.3	5.5	3.4	6.4	-	-	2.1	4.3	2.5	-	8.9	11.5	-	3.8	4.5	5.2	4.2	8.2	1.1	3.8
58	Cycleisosativene	1378	1345-1406	1483	1470-1492	-	-	-	-	1.7	-	-	-	-	0.8	-	-	-	-	-	-	-	-	-	-
59	β-Elemene	1391	1366-1406			1.7	1.2	4.4	4.7	-	-	-	1.1	-	-	0.6	-	1.2	0.7	3.2	-	-	-	-	0.9
60	α-Gurjunene	1418	1408-1434			-	1.0	-	-	-	-	-	-	-	-	-	-	-	-	-	-	-	-	-	-
61	β-Ylangene	1420	1405-1425	1591	1547-1586	-	-	-	-	-	-	-	-	0.6	-	1.7	-	-	-	-	1.8	2.4	-	-	-
62	α-Santalene	1420	1412-1420			-	0.5	-	-	-	-	-	-	-	-	-	-	-	-	-	-	-	-	-	-

63	Caryophyllene	1428	1418-1420	1591	1589-1617	6.3	7.6	10.8	8.6	-	-	0.7	8.2	2.8	-	13.8	12.6	5.8	24.6	6.0	2.5	2.8	7.3	5.7	9.9
64	Isocaryophyllene	1429	1419-1434	1577	1582-1589	-	-	0.7	-	4.9	-	4.5	-	-	-	-	-	-	-	-	-	-	-	-	-
65	E-α-Bergamotene	1430	1431-1438			-	-	-	-	-	-	-	-	-	-	1.3	-	-	-	-	-	-	-	-	-
66	γ-Elemene	1430	1418-1482			-	-	0.5	-	-	-	-	-	-	-	-	-	-	-	-	-	-	-	-	-
67	β-Copaene	1430	1426-1442			0.5	0.7	-	1.0	-	-	0.8	-	-	-	1.7	-	-	-	-	-	-	-	-	0.5
68	α-Guaiene	1444	1436-1444	1604	1589-1600	0.7	0.7	-	-	-	-	-	-	0.6	2.5	-	-	-	-	-	-	2.0	-	-	-
69	Alloaromadendrene	1450	1451-1471	1650	1604-1662	0.5	-	-	3.1	-	-	-	-	-	0.6	4.0	-	-	-	-	-	-	-	0.6	0.7
70	Selina-5,11-diene	1453	1446-1447			-	-	-	6.9	-	-	-	-	-	-	-	-	-	-	-	-	-	-	-	-
71	E-β-Ionone	1457	1459-1485			-	-	-	2.5	-	-	-	-	-	-	-	-	-	-	-	-	-	-	-	-
72	Humulene	1462	1451-1477	1675	1641-1705	0.7	0.8	1.5	3.9	-	-	2.6	3.8	-	-	1.9	2.4	0.6	1.5	0.8	-	0.5	0.8	2.5	1.8
73	γ-Gurjunene	1469	1465-1476			-	-	-	1.1	-	-	-	-	-	-	-	-	-	-	-	-	-	-	-	-
74	Germacrene D	1476	1474-1503	1716	1687-1729	7.0	7.0	8.9	5.6	1.3	-	2.4	5.1	1.0	-	1.9	14.0	11.0	0.9	1.1	0.9	3.8	8.4	2.8	10.8
75	Bicyclosesquiphellandrene	1477	1470-1488			-	-	-	0.6	-	-	-	-	-	-	-	-	-	-	-	-	-	-	-	-
76	γ-Muurolene	1477	1449-1491			0.5	0.5	-	1.2	-	-	0.9	1.2	-	-	-	1.0	-	1.4	-	-	-	0.9	0.6	0.6
77	Isogermacrene D	1478	1446-1501			-	-	-	-	-	-	-	-	-	-	-	-	0.7	-	-	-	-	-	1.2	-
78	β-Selinene	1478	1476-1493			-	-	-	-	1.8	-	-	-	-	-	-	-	-	-	-	-	-	-	-	-
79	Cadina-1(6),4-diene	1479	1472-1481			-	0.7	2.1	5.1	-	-	-	4.7	2.4	-	3.7	6.3	-	-	1.7	1.1	-	-	-	-
80	α-Muurolene	1480	1469-1497			-	-	-	-	-	-	0.6	-	-	-	-	-	-	-	-	-	-	-	-	-
81	Germacrene D-4-ol	1480	1571-1576			2.3	-	-	-	-	-	-	-	-	-	-	-	-	-	-	-	-	1.5	-	1.2
82	γ-Cadinene	1483	1480-1534			0.6	0.6	-	2.4	-	-	0.6	1.0	-	-	-	-	-	0.8	-	-	-	-	0.8	-
83	Guaia-1(10),11-diene	1490	1455-1525			-	0.6	-	-	-	-	-	-	-	-	-	-	-	0.6	-	-	-	-	-	-
84	α-Ylangene	1491	1474-1493			-	1.2	-	0.7	-	-	-	0.6	-	-	-	-	-	-	-	-	-	-	-	-
85	E-Cadina-1(10).4-diene	1493	1502-1517			-	-	-	-	-	-	1.6	0.6	-	-	-	-	0.6	-	-	-	-	-	1.8	-
86	Bicyclogermacrene	1494	1495-1518			-	-	0.6	-	-	-	-	-	-	-	-	-	-	-	-	-	-	-	2.6	-
87	γ-Amorphene	1496	1480-1500			-	-	-	-	-	-	1.8	-	-	-	-	-	-	-	-	-	-	0.6	-	-
88	Eudesma-4(14),11-diene	1497	1472-1517			-	-	1.9	1.3	-	-	-	1.4	-	1.2	5.0	-	-	-	-	-	-	1.6	-	1.1
89	Curcumene	1502	1504-1524			-	-	-	-	-	-	1.1	-	-	-	-	-	-	-	-	-	-	-	-	-
90	α-Cadinene	1512	1509-1530			2.6	2.4	-	-	-	-	-	-	-	0.6	-	-	-	-	-	-	-	-	-	-
91	Myristicin	1513	1516-1526	2279	2225-2272	22.9	5.6	-	-	-	0.7	-	-	-	-	-	-	-	-	-	-	64.2	-	-	9.2
92	β-Guaiene	1523	1504-1533			-	-	-	0.9	-	-	-	-	-	0.8	-	-	-	-	-	-	-	-	-	-
93	σ-Cadinene	1524	1509-1530			-	-	-	-	0.8	-	-	-	-	-	-	-	-	-	-	-	-	-	-	-
94	α-Farnesene	1526	1504-1522			-	-	-	-	-	-	-	1.7	-	-	0.6	-	-	-	-	-	-	-	-	-
95	Z-Calamenene	1534	1537-1537			-	-	-	-	-	-	-	-	-	-	-	-	-	1.6	-	-	-	-	-	1.0
96	Germacrene B	1545	1543-1556			4.2	7.2	11.0	4.7	-	-	-	4.6	1.0	-	4.5	3.8	3.9	4.2	3.1	2.5	-	3.7	-	4.3
97	Elemicin	1552	1521-1554	2228	2226-2246	-	-	-	-	-	-	-	-	-	-	-	-	1.1	-	-	-	-	-	-	0.9
98	E-Nerolidol	1564	1538-1565	2048	1998-2074	1.0	0.7	11.8	-	-	-	-	1.1	6.5	1.6	0.8	4.4	0.7	-	0.9	-	-	-	-	5.4
99	Ylangenol	1574	1574-1666			1.1	0.6	-	0.5	-	-	-	-	-	-	1.1	-	-	1.3	-	-	-	-	-	-
100	Spathulenol	1575	1569-1603	2138	2138-2154	1.0	2.0	4.2	3.0	-	-	-	0.6	4.6	1.0	0.7	1.0	3.0	-	-	-	-	-	-	-

101	Globulol	1576	1560-1623	2103	2070-2104	-	-	-	-	-	-	0.8	-	-	0.7	0.9	-	-	1.0	-	-	-	-	1.9	-
102	δ-Cadinol	1580	1576-1644			-	-	-	1.0	-	-	-	-	-	-	-	-	-	-	-	-	-	-	-	-
103	α-Copaene-4-ol	1585	1588-1604			-	-	-	-	-	-	-	-	-	-	-	-	-	-	-	-	-	-	0.6	-
104	Viridiflorol	1590	1572-1587			-	4.1	-	-	-	-	-	3.1	-	-	-	0.7	-	1.3	-	-	-	-	-	-
105	Eudesm-4(14)-en-11-ol	1593	1543-1605			-	-	-	1.5	-	-	-	0.8	-	-	-	-	-	10.7	-	-	-	-	-	2.0
106	Caryophyllene oxide	1593	1573-1606			1.1	1.7	2.0	1.3	-	-	0.91	1.5	1.1	4.4	1.9	3.0	0.7	6.1	0.9	-	0.5	2.0	0.8	1.6
107	Guaiol	1595	1588-1605			-	-	-	0.5	-	-	-	0.7	7.6	-	-	-	-	-	-	-	-	-	-	-
108	δ-cedrol	1596	1589-1604			0.6	-	-	-	-	-	-	-	-	0.6	-	-	-	-	-	-	-	-	-	-
109	Humulene 6,7-epoxide	1608	1602-1610			-	-	-	1.5	-	-	0.7	-	-	-	-	-	-	-	-	-	-	-	-	-
110	γ-Eudesmol	1626	1614-1621																4.8						
111	Epicubenol	1627	1623-1631			0.5	-	-	0.89	-	-	-	0.5	-	-	-	0.7	-	2.7	-	-	-	-	-	0.8
112	τ-Muurolol	1628	1613-1661	1285	2143-2209	0.6	-	-	-	-	-	0.7	-	-	-	-	0.7	-	-	-	-	-	0.7	-	-
113	Muurola-4,10(14)-dien-1-β-ol	1634	1620-1663			-	-	-	-	0.5	-	-	-	-	-	-	-	-	-	-	-	-	-	-	-
114	Dilapiol	1634	1620-1625	2362	2305-2384	29.5	23.3	0.8	-	-	-	-	7.3	0.8	-	-	2.1	22.0	-	-	0.6	-	1.4	-	-
115	α-Cadinol	1653	1603-1677	2244	2221-2256	1.1	-	-	1.3	-	-	-	0.5	-	-	-	0.8	-	-	-	-	-	-	-	0.6
116	τ-Cadinol	1654	1638-1644	2145	2139-2197	-	-	-	-	-	-	-	0.5	-	-	-	-	-	-	-	-	-	0.7	-	-
117	β-Bisabolol	1662	1665-1683			-	-	-	-	-	-	0.6	-	-	-	-	-	-	-	-	-	-	-	-	-
118	Apiole	1679	1674-1682	2450	2431-2460	-	7.3	2.9	0.7	-	-	2.8	6.9	0.6	-	3.8	3.0	37.2	-	-	-	-	3.4	-	13.9
119	Farnesyl alcohol	1710	1682-1703				-	-	-	-	-	2.4		-	-	-	-	-	14.7	-	-	-	-	2.1	-
120	ent-Germacra4(15),5,10(14)-trien-1β-ol	1756	1701-1747			1.8	-	0.6	1.3	-	-	1.8	-	0.6	-	-	-	-	0.8	-	-	-	-	1.0	0.7
121	Cembrene	1940	1929-1939			-	-	-	-	-	-	-	-	-	-	0.6	-	-	3.5	-	-	-	-	-	2.9
122	Arachidonate	2231	2254-2294.7			-	-	1.0	-	-	-	-	-	1.0	-	0.6	1.4	-	-	-	-	-	-	-	0.8
	Hydrocarbon-type monoterpenoids					1.6	12.8	19.4	12.0	18.2	23.4	25.2	24.1	51.8	47.3	27.9	19.4	6.1	2.3	5.4	13.0	14.2	50.6	52.8	14.9
	Oxygenated monoterpenoids					0.6	0.7	0	8.5	70.4	10.1	40.2	9.7	0.7	33.8	4.5	3.4	0.0	0	0.0	0.0	0	2.3	13.8	0.0
	Hydrocarbon-type sesquiterpenoids					28.0	35.3	45.6	60.2	10.5	0.0	21.0	38.6	11.5	4.3	48.4	53.1	23.8	47.3	20.1	14.1	15.7	31.5	22.6	39.1
	Oxygenated sesquiterpenoids					11.7	12.0	19.3	14.8	0.5	0.0	6.7	9.3	19.7	8.3	5.4	11.2	4.4	42.6	1.8	0	0.5	4.9	4.3	12.4
	Phenylpropanoids					52.5	38.5	7.5	1.4	0.0	65.0	0.0	14.2	8.9	0.0	4.5	5.6	60.3	0.0	66.1	67.9	64.2	4.7	0.0	23.1
	Others					0.0	0.0	2.1	0.0	0.0	0.0	2.7	0.0	1.1	1.4	4.7	1.4	0.5	0.7	0	0.6	0.0	0.0	2.1	5.4
	**TOTAL**					**94.3**	**96.9**	**94.0**	**96.8**	**99.5**	**98.5**	**95.7**	**95.8**	**93.5**	**95.2**	**95.3**	**93.6**	**95.1**	**92.8**	**93.4**	**95.6**	**94.5**	**94.0**	**95.3**	**94.8**

RI: Retention index (**a**) DB-5MS not polar column. (**b**) HP-INNOWax polar column

4. Statistical analysis post hoc Tukey test bioactive AEs

IC50

	EOs	N	Subset for alpha = 0.05												
			1	2	3	4	5	6	7	8	9	10	11	12	13
HSD Tukey[a]	A18	3	,5800												
	A2	3	,6100												
	A1	3	,6200												
	A29	3	2,0500												
	A11	3		12,5400											
	A7	3		15,0300											
	A34	3			22,7100										
	A10	3				35,4500									
	A30	3					41,5700								
	A5	3					44,8800								
	A14	3						54,7600							
	A23	3							60,1400						
	A21	3							61,8700						
	A31	3								82,7300					
	A17	3								85,6100	85,6100				
	A12	3									89,1700	89,1700			
	A3	3										90,2000			
	A4	3											125,7900		
	A13	3												150,1300	
	A20	3													184,2700
	Sig.		,999	,840	1,000	1,000	,401	1,000	,994	,642	,282	1,000	1,000	1,000	1,000

The means for the groups in the homogeneous subsets are displayed.

a. Use the harmonic mean sample size = 3,000.

Figure S1. Table of results of the Tukey test EOs. Group means for homogeneous subsets are displayed. Confidence level $\alpha = 0.05$.

a. Uses the harmonic mean sample size = 4.0

5. Chemical characterization of the compound Piperitone (Tables S4 and S5, Figures S2, S3 and S4)

Isolation of piperitone (C34) from the essential oil of *Piper asperisculum*: The essential oil of *P. asperisculum* (1.0 g) was subjected to column chromatography (CC) using isocratic elution with a hexane : DCM (6 : 4) mixture, resulting in six fractions. Fractions 2 and 3 were combined and subjected to further CC using a hexane:DCM (7:3) mixture, yielding a light yellow viscous liquid (245.7 mg) corresponding to piperitone C34.

Table S4. Spectroscopic data and NMR spectra of compound **C34.** [86]

<table>
<tr><td rowspan="4">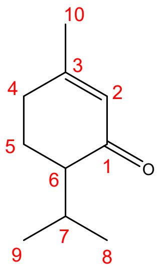
</td><td>Name</td><td>Piperitone</td></tr>
<tr><td>Physical state</td><td>Pale yellow oily liquid</td></tr>
<tr><td>Molecular formula</td><td>$C_{10}H_{16}O$</td></tr>
<tr><td>Molecular weight</td><td>152.24 g/mol</td></tr>
<tr><td colspan="3">RMN ^{1}H: (400 MHz, $CDCl_3$) δ_H (ppm) 5.81 (q, J = 1.3 & 1.5 Hz, 1H, H-2), 2.34 (m, 1H, H-4α), 2.28 (m, 1H, H-4β), 2.28 (m, 1H, H-6), 1.98 (m, 1H, H-7), 1.90 (s, 3H, H-10), 1.98 (m, 1H, H-5α), 1.79 (m, 1H, H-5β), 0.92 (d, J = 7.0 Hz, 3H, H-8), 0.83 (d, J = 6.9 Hz, 3H, H-9).</td></tr>
<tr><td colspan="3">RMN APT: (100 MHz, $CDCl_3$) δ_C (ppm) 201.4 (C-1), 161.2 (C-3), 126.9 (C-2), 51.7 (C-6), 30.5 (C-4), 25.9 (C-7), 24.2 (C-10), 23.1 (C-5), 20.8 (C-9), 18.6 (C-8).</td></tr>
</table>

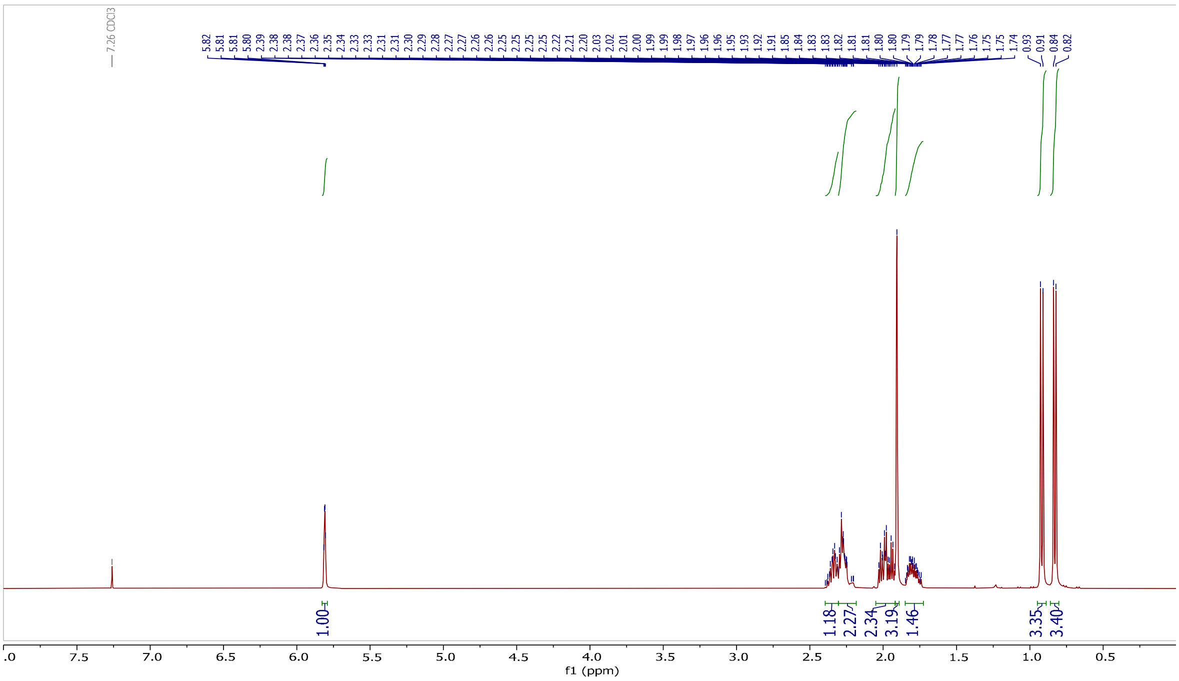

Figure S2. NMR ^{1}H spectrum **C34** (CDCl$_3$, 400 MHz)

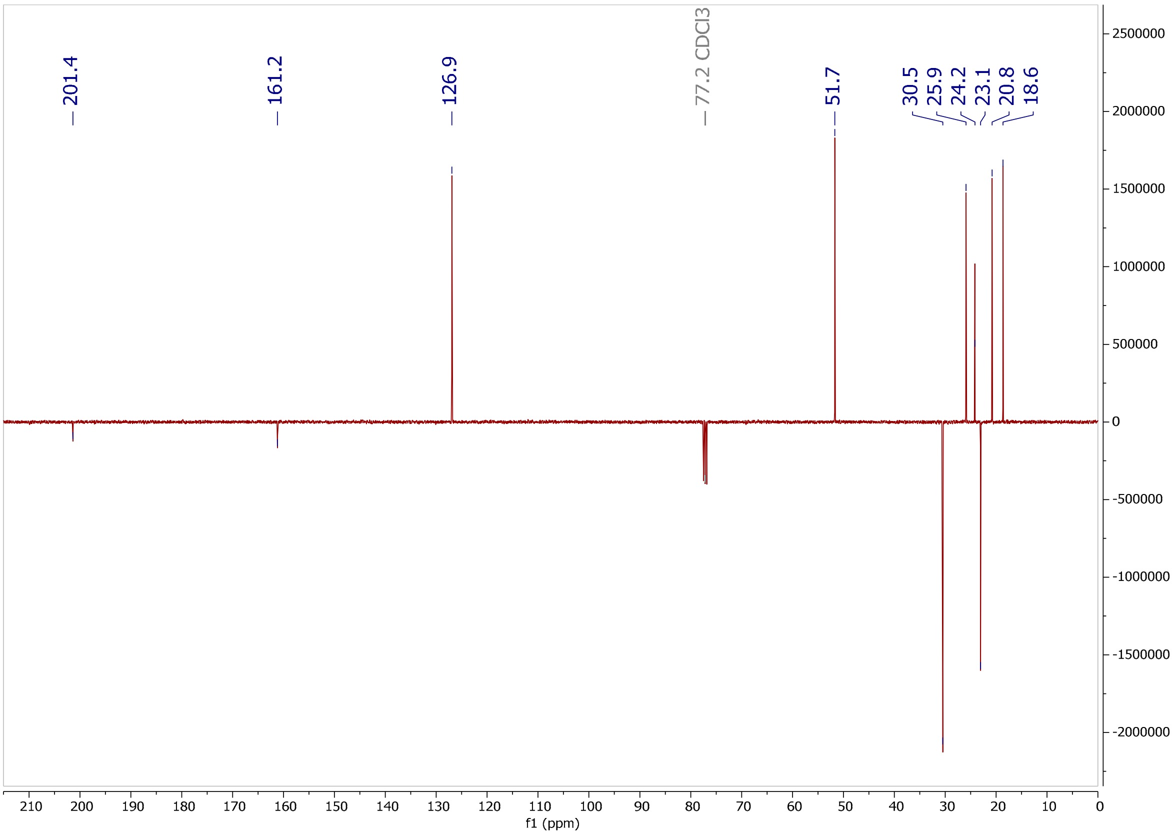

Figure S3. NMR APT spectrum (CDCl$_3$, 100 MHz)

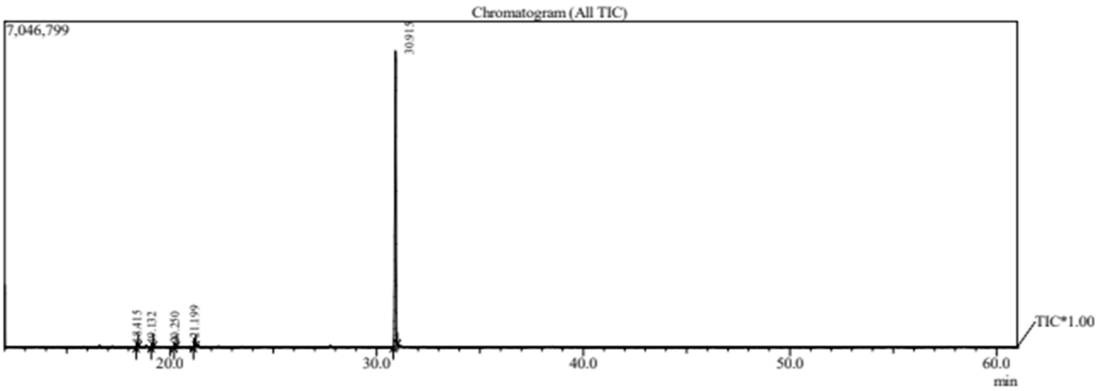

Figure S4. Chromatographic profile (TIC) from GC-MS analysis (DB-5MS) of piperitone isolated from *Piper asperisculum*.

Table S5. Chemical composition of piperitone isolated from *Piper asperisculum* (DB-5MS).

rt	Area (%)	Exp LRI.	Ref LRI	Compound
18.415	1.07	995	998-1097	7-octenone
19.132	0.81	1015	960-990	β-Pinene
20.250	0.9	1015	985-1011	α-Phellandrene
21.199	2.29	1031	1023-1038	D-Limonene
30.915	94.93	1258	1250-1256	Piperitone

6. Tukey's post hoc test analysis of the compounds evaluated.

IC50

	Compound	N	Subset for alpha= 0.05										
			1	2	3	4	5	6	7	8	9	10	11
HSD Tukey[a]	C28	3	,4700										
	C14	3	,5500										
	C3	3	3,6600	3,6600									
	C11	3	3,9900	3,9900									
	C38	3	4,2700	4,2700									
	C25	3	4,8400	4,8400									
	C26	3	6,5800	6,5800	6,5800								
	C10	3	7,2500	7,2500	7,2500								
	C40	3		12,2500	12,2500	12,2500							
	C36	3			15,0300	15,0300	15,0300						
	C13	3				17,4400	17,4400						
	C29	3					21,9600	21,9600					
	C9	3					22,4400	22,4400					
	C18	3					22,8700	22,8700					
	C34	3						28,0600					
	C15	3							41,4800				
	C2	3							42,1600				
	C39	3							42,7000				
	C23	3							45,1800				
	C1	3								59,6500			
	C30	3								60,2400			
	C22	3									73,2000		
	C35	3									75,6400		
	C16	3										85,4200	
	C24	3										85,4700	
	C17	3										89,4200	
	C37	3											103,7000
	C31	3											109,8000
	Sig.		,396	,070	,082	,860	,157	,607	,997	1,000	1,000	,991	,607

The means for the groups in the homogeneous subsets are displayed.

a. Use the harmonic mean sample size = 3,000.

Figure S5. Table of results of the compound Tukey test. Group means for homogeneous subsets are displayed. Confidence level $\alpha = 0.05$.

a. Uses the harmonic mean sample size = 4.0
